# Assessing transmission attribution risk from simulated sequencing data in HIV molecular epidemiology

**DOI:** 10.1097/QAD.0000000000003820

**Published:** 2024-03-04

**Authors:** Fabrícia F. Nascimento, Sanjay R. Mehta, Susan J. Little, Erik M. Volz

**Affiliations:** a MRC Centre for Global Infectious Disease Analysis and the Department of Infectious Disease Epidemiology, Imperial College London, London, UK; b Division of Infectious Diseases, University of California San Diego, San Diego, CA, USA.

**Keywords:** consensus sequences, HIV, molecular epidemiology, next-generation sequencing, phylogenetics, source attribution

## Abstract

**Background:**

HIV molecular epidemiology (ME) is the analysis of sequence data together with individual-level clinical, demographic, and behavioral data to understand HIV epidemiology. The use of ME has raised concerns regarding identification of the putative source in direct transmission events. This could result in harm ranging from stigma to criminal prosecution in some jurisdictions. Here we assessed the risks of ME using simulated HIV genetic sequencing data.

**Methods:**

We simulated social networks of men-who-have-sex-with-men, calibrating the simulations to data from San Diego. We used these networks to simulate consensus and next-generation sequence (NGS) data to evaluate the risks of identifying direct transmissions using different HIV sequence lengths, and population sampling depths. To identify the source of transmissions, we calculated infector probability and used phyloscanner software for the analysis of consensus and NGS data, respectively.

**Results:**

Consensus sequence analyses showed that the risk of correctly inferring the source (direct transmission) within identified transmission pairs was very small and independent of sampling depth. Alternatively, NGS analyses showed that identification of the source of a transmission was very accurate, but only for 6.5% of inferred pairs. False positive transmissions were also observed, where one or more unobserved intermediaries were present when compared to the true network.

**Conclusion:**

Source attribution using consensus sequences rarely infers direct transmission pairs with high confidence but is still useful for population studies. In contrast, source attribution using NGS data was much more accurate in identifying direct transmission pairs, but for only a small percentage of transmission pairs analyzed.

## Introduction

Molecular epidemiology (ME) is the analysis of pathogen genetic sequence data combined with individual-level clinical, demographic, and behavioral data to understand the epidemiology of a disease [1]. HIV ME has emerged as a tool to identify risk factors associated with linked transmission events within and between different populations [2]. ME can answer questions at different scales, such as ‘Where is HIV transmission occurring at the greatest rate?’; ‘Which groups are at greatest risk of transmitting and acquiring infection?’; and ‘How are transmission patterns likely to change in the future?’. However, the use of ME has raised ethical concerns about whether such analyses could identify individuals linked by a direct transmission (i.e., person A transmitted to person B without intermediate individuals) or transmission pairs (i.e., person A transmitted to person B or person B transmitted to A without intermediate individuals).

Attribution of a direct HIV transmission to an individual may have harmful consequences, ranging from stigma to criminal prosecution (in jurisdictions with laws criminalizing HIV transmission and nondisclosure) [1]. For example, ME analysis using *pol* and *env* were used in two criminal cases to implicate defendants as the source of transmission [3]. However, in these criminal cases, the analyses based on gene sequences was only one piece of evidence, which in isolation was not sufficient to establish transmission [3].

Identification of transmission pairs with ME using partial *pol* sequences generated by Sanger sequencing (consensus sequence data) with high credibility is seldom possible [4]. However, with the increasing availability and reduced cost of next-generation sequencing (NGS) technologies, it is possible to sequence full-length HIV genomes and sample the viral diversity, increasing the potential to correctly identify transmission pairs [5–8].

The risks associated with identifying direct transmission pairs have the potential to limit use of ME and limit the willingness of people who are vulnerable to HIV from seeking HIV testing, since HIV sequence data is routinely collected to guide the use of antiretroviral treatment (ART). These sequence data are also reported to public health departments for epidemiological surveillance. Despite the potential risks associated with HIV ME, qualitative work to understand ME perceptions among stakeholders found general support for HIV ME, with most feeling that the potential benefits to public health outweighed the risks [9]. However, there were still concerns about breaches of privacy and a potential increase in antigay sentiment [9].

The objective of our paper was to use simulated transmission networks, consensus sequences and NGS to quantify and contrast the source attribution risks of HIV ME between both sequencing technologies. In contrast to previous reports [5,6,10,11], we used random samples and sampling times to understand whether we could use ME to infer direct transmissions in a surveillance setting.

## Methods

Methods are summarized here. For a complete description see Supplementary Material.

## Network simulations

To simulate HIV transmission dynamics, we used the R package EpiModel [12] to implement an agent-based framework. Agent-based simulations allow tracking of individuals through time, such as the formation of sexual relationships and HIV transmission events. Our simulated HIV dynamics were based on a simple mathematical model for HIV transmissions in men-who-have-sex-with-men (MSM) parameterized by epidemiological surveillance data from San Diego, USA [13].

Our transmission dynamics model included five stages of HIV infection, defined as acute and early HIV; three stages of chronic infection; and AIDS [14]. Our model also included three stages of diagnosis defined as undiagnosed; diagnosed and not on ART; and diagnosed and on ART. A novel feature of our model was the incorporation of migration events in which individuals were allowed to migrate between two different populations, here referred to as *region* and *global*. *Region* was representative of the San Diego MSM population, while *global* was representative of a bigger population that would account for importation of lineages from a global reservoir. For HIV evolution, migration allowed the analysis of lineages which had a common ancestor in *global* predating the epidemic expansion in *region*.

### Model parameters

The parameters used in our agent-based simulations described the initial population size and structure; migration in and out of San Diego; transmission probabilities; and probability an MSM would be diagnosed and start ART over time (Table S1, Supplemental Digital Content). Transmission probabilities varied based on stage of infection, diagnosis, and care. Individuals with acute and early HIV, undiagnosed, or diagnosed but not receiving ART had a higher probability of transmission than individuals diagnosed with chronic HIV and on ART [15].

Because real epidemics typically feature super-spreading and because this can have a large influence on genetic diversity of a pathogen [16], the population was further stratified into two risk categories with 20% of individuals assigned to a high-risk group, associated with a 10-fold higher rate of HIV transmissions compared to the individuals in the low-risk group [15].

### Model calibrations

**Fig. 1:**
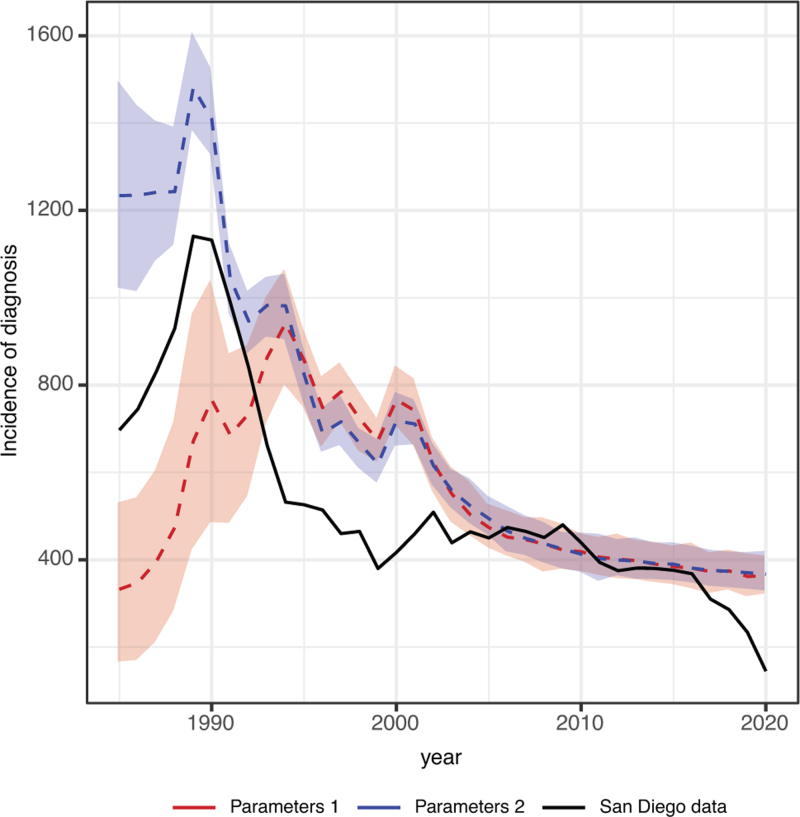
Incidence of diagnosis.

To calibrate our network simulations, we used random Latin hypercube sampling [17] to sample the parameters we could not obtain from San Diego surveillance data, such as the initial number of HIV infected individuals and the probability an MSM would initiate ART (Table S2, Supplemental Digital Content). After selecting the best fit simulated trajectory for HIV incidence of diagnosis, we only ran 30 replicates using the best two parameter group values (Fig. 1), because our simulations required significant computational resources including storage space.

Absolute numbers for incidence of diagnosis (*y*-axis) from 1980 to 2020 (*x*-axis) for the two best combination of parameter values, referred to as parameters 1 and parameters 2, that showed the best fit to the surveillance data of incidence of diagnosis for MSM in San Diego (see Table S6, Supplemental Digital Content). Medians and 95% credible interval are shown as dashed line and shaded area, respectively. Black solid line represents the incidence of diagnosis for surveillance data for San Diego.

### Output of simulations

For each network simulation, we obtained a transmission matrix containing the time of transmission, the identification number (ID) of individuals attributed as the source and recipient of the HIV transmission, and the origin (*region* or *global*) of each ID at the time of transmission. We also recorded additional metadata for each individual at each simulation run and time step, such as the HIV stage of infection, and start of ART. These metadata were used in the phylogenetic and source attribution analyses described below.

## Simulation and analysis of genetic sequences

**Fig. 2:**
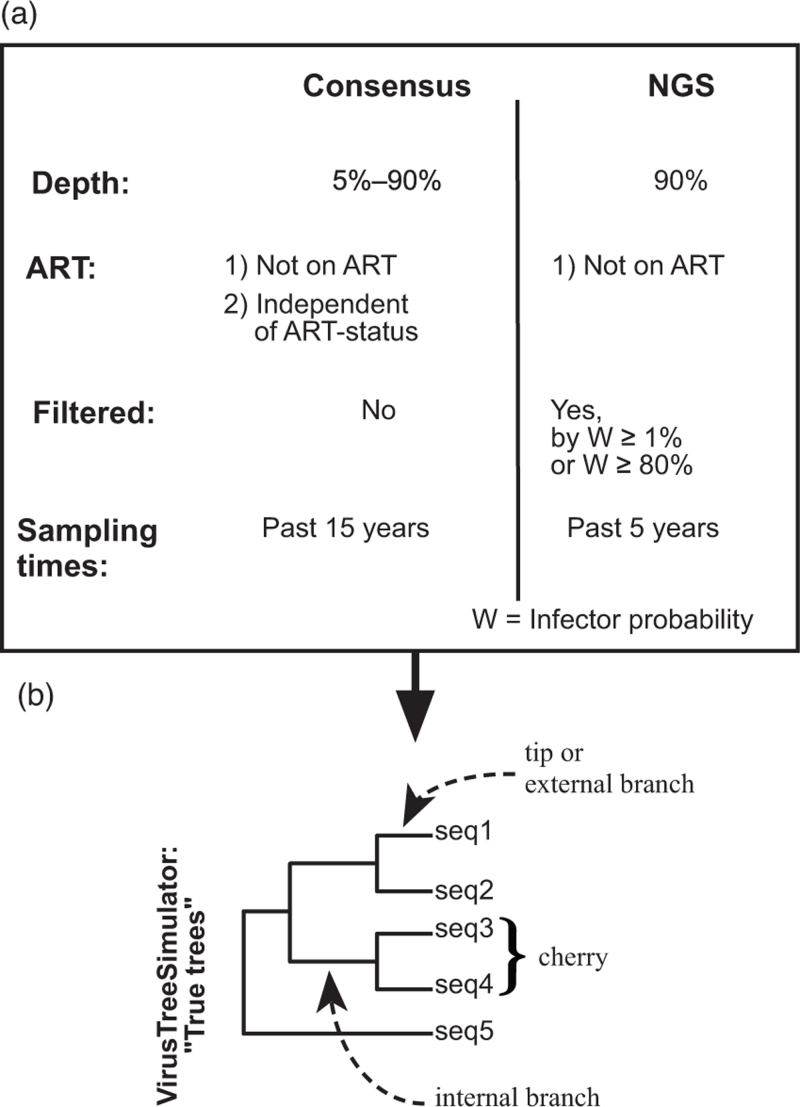
Sampling strategy and phylogenetic trees.

A summary of the sampling strategies used to simulate consensus sequences and NGS is provided (Fig. 2). Due to the significant computational resources required for NGS simulation, we were unable to use the same strategy as used for the consensus sequences.

(a) Schematic illustration of the sampling strategies used to random sample individuals and sampling times to (b) estimate the ‘true trees’ using the VirusTreeSimulator program. The illustration in (b) shows the main parts of a phylogenetic tree discussed in the text. For example, seq1 and seq2 forms a cherry as well as seq3 and seq4. For consensus sequences, we only analyzed tips that formed a cherry. Depth = the sampling population size of diagnosed individuals; ART = antiretroviral treatment; Filtered = whether a filtering strategy was implemented on the total number of sampled individuals; and sampling times = the time that virus was sampled from an individual.

### Consensus sequences

We carried out two different sampling strategies from the transmission matrix: We sampled individuals who were active (have not departed the network), diagnosed, and both not on ART at the time of sampling and independent of ART-status at the time of sampling. We then randomly chose dates in the past 15 years (time of sampling) and varied the percentage of individuals sampled from 5% to 90% (Fig. 2).

We used the sampled individuals and sampling times together with the transmission matrix to obtain phylogenetic trees using the program VirusTreeSimulator (https://github.com/PangeaHIV/VirusTreeSimulator). We referred to these trees as *true trees*. In the real world, true trees are not known a priori but must be inferred based on viral genetic sequences. We used the true trees with Seq-Gen v1.3.4 [18] to simulate genetic sequence alignments of 1000 base pairs (bp) (equivalent to the partial *pol* gene), and 10 000 bp (equivalent to the HIV whole-genome). For each alignment, we estimated phylogenetic trees using maximum likelihood (ML) with the program IQ-TREE v2.2.0 [19] followed by estimation of time-scaled trees using *treedater* v0.5.3 [20].

For the source attribution, we calculated the infector probability (*W*) which is the probability that a particular host *i* infected a particular host *j*[4]. This can be estimated using a combination of time-scaled trees and epidemiological surveillance data including recency of infection [21–23]. We used the method by Volz and Frost [4] which incorporates incidence and prevalence data to adjust for incomplete sampling and the fact that unsampled individuals can be an unobserved source of infection [21].

We calculated *W* for the true trees and to account for error when inferring phylogenetic trees, we also calculated *W* for the time-trees estimated with 1000 bp and 10 000 bp simulated sequence alignments, and used the same values for incidence, prevalence, CD4 count, and recency test as obtained in the network simulations.

To evaluate the accuracy of estimated *W* we calculated precision-recall curves (PRC) for all pairs of adjacent tips in a phylogeny (also known as a cherry) (Fig. 2). The PRC was computed for cherries instead of all pairs within the sample because this would flood the results with true negatives, allowing our prediction to appear overly accurate as distant pairs in the tree will have very low *W* values [4]. Furthermore, when imbalanced data is analyzed, such as described here in which we had very few positive classes compared to negative classes, a PRC provides more useful interpretation of the data than a receiver operating characteristic (ROC) curve [24].

To evaluate whether a pair represented a transmission pair, we compared *W* results with the network simulations, where the ground truth is known, and we were able to quantify the true positives (TP), false positives (FP), true negatives (TN) and false negatives (FN). PRC was computed using merged data from 30 replicates per parameter group values to estimate the recall and precision over a range of population sample sizes. We also estimated the area under the curve (AUC) for each PRC as a measure to understand *W* performance in identifying transmission pairs (independent of source attribution).

### Next-generation sequencing

We carried out one sample strategy in which we used the transmission matrix to sample 90% of individuals who were active, diagnosed and not on ART at the time of sampling (Fig. 2). We then randomly sampled a date in the past 5 years (time of sampling). We used the sampled individuals and dates together with the transmission matrix to obtain phylogenetic trees using the program VirusTreeSimulator. We simulated 10 proviruses for each individual to mimic viral diversity.

To decrease the amount of disk space required for NGS simulation, we derived a consensus tree by removing all but leaving one viral sequence per individual, and estimated *W*. We then analyzed a dataset containing all pairs that showed *W*≥80% and a second dataset containing all pairs that showed *W*≥1%.

We simulated genomic sequence alignments using Seq-Gen [18] for all pairs that showed *W* ≥80% or *W* ≥1%. We used the program ART_Illumina v2.5.8 [25] to simulate 250 bp Illumina paired-end reads (Table S3, Supplemental Digital Content) and used the program SHIVER [26] to map the simulated Illumina reads to a HIV-1 subtype B reference sequence (GenBank accession number: K03455).

For source attribution, we used phyloscanner [27] to infer transmission pairs using the mapped reads and previously reported parameter values [6] (Supplementary Material; Tables S4 and S5, Supplemental Digital Content). To evaluate the accuracy of phyloscanner to identify a transmission pair we compared the results obtained with phyloscanner and the ground truth network simulations and quantified the total number of pairs identified as TP, FP, TN, and FN. Using these values, we estimated sensitivity, specificity, and precision for each parameter group values.

We also calculated PRC for phyloscanner results filtering by *W* ≥1% and the AUC for each PRC as a measure to understand the performance of phyloscanner to identify transmission pairs.

## Software and code availability

We developed an R package HIVepisim (https://github.com/thednainus/HIVepisim) to simulate HIV epidemics based on EpiModel [12]. We uploaded the code used to generate our results at https://github.com/thednainus/HIVepisimAnalysis.

## Computational resources

Analyses were carried out using the Imperial College Research Computing Service; the Open Science Grid [28]; and the bridges2 system at the Pittsburgh Supercomputing Center available through the Extreme Science and Engineering Discovery Environment [29].

## Results

### Incidence of diagnosis

Figure 1 shows the simulated trajectories for incidence of HIV diagnoses obtained with the two best parameter group values (Table S6, Supplemental Digital Content). These simulations differed substantially before 1995, after which both trajectories were very similar, with parameter group 1 trajectory showing a larger credible interval than parameter group 2 (Fig. 1).

### Analysis of consensus sequences

Each individual viral genetic sequence is represented by a tip of the phylogenetic tree (Fig. 2), and because we sampled different population depths, we analyzed a smaller number of total pairs (Tables S2.3–S2.14, [30]) for a 5% sampling depth compared to 90%. The main difference between sampling strategy 1 (diagnosed individuals not on ART) and strategy 2 (diagnosed individuals independent of ART-status) was that the total number of pairs analyzed was larger for strategy 2.

To understand whether we could use infector probabilities to identify transmission pairs, we generated PRCs. PRCs showed that infector probabilities performed better than a random classifier, which is indicated by the horizontal lines (Fig. 3a and b). In general, infector probability performance was similar independent of migration rates and sampling depth (Figures S2–S13, Supplemental Digital Content).

**Fig. 3:**
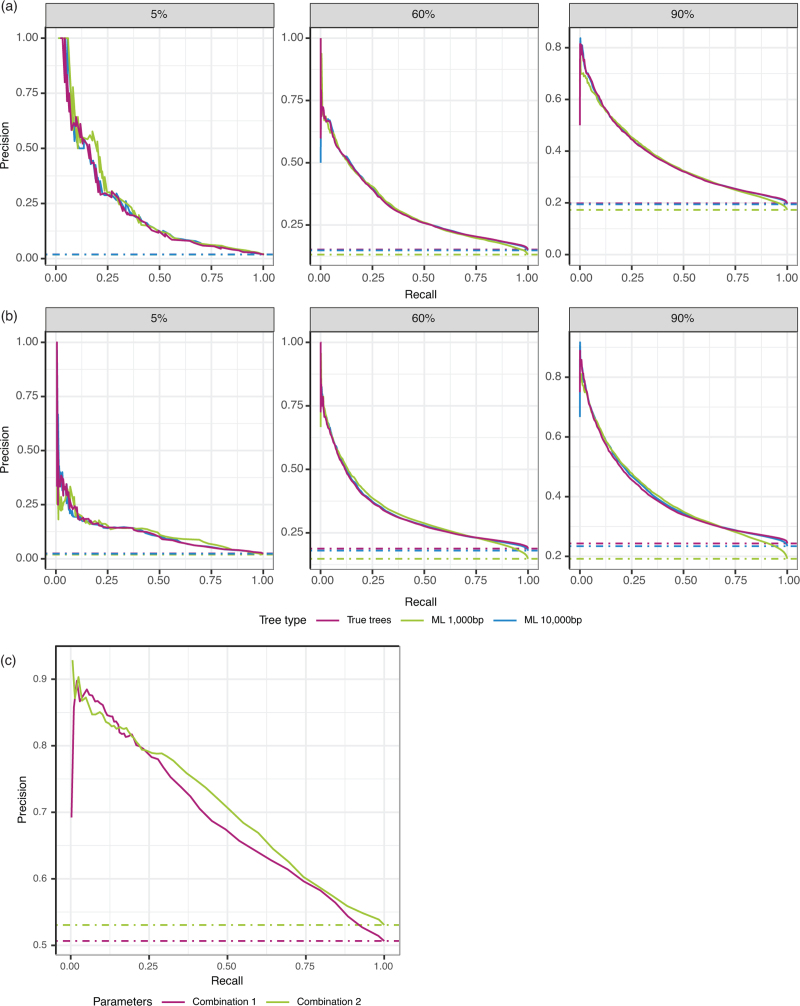
Precision–recall curves (PRC).

PRC for consensus sequences for sampling depth of 5%, 60% and 90% of the diagnosed individuals not on ART for adjacent tips (cherry or 2-clades; Fig. 2) in the phylogenetic tree for (a) parameter group 1 and (b) parameter group 2. PRC for phyloscanner results filtered by *W***≥**1% of the diagnosed individuals not on ART for parameter groups 1 and 2 (c). All PRCs are for a total of 500 migrants to and from *region* per year. For definition of parameter group 1 and group 2, see Fig. 1 and Table S6, Supplemental Digital Content. *y*-axis shows precision = TP/(TP + FP); while *x*-axis shows the recall = TP/(TP + FN). TP = total number of pairs identified as true positives; FP = total number of pairs identified as false positives and FN = total number of pairs identified as false negatives. Horizontal lines represent a random classifier.

There is no guideline for selecting the optimal *W* value to identify transmission pairs. To demonstrate the impact of varying *W* values on our ability to identify transmission pairs, we assessed sensitivity, specificity, and precision at *W* thresholds of 80% and 90% (Table 1 and Tables S8–S19, Supplemental Digital Content). We observed that sensitivity decreased for the 90% threshold because the number of TP were smaller than the 80% threshold. In contrast, precision increased for the 90% threshold because the number of TP and FP were smaller than for the 80% threshold (Tables S2.3–S2.14, [30]). When checking whether we could attribute the source of a direct transmission among TP pairs, we observed that for a 5% population depth, we correctly identified direct transmissions in 100% of the pairs. However, this represented only two to seven pairs from the total analyzed. As we increased sampling depth, we identified direct transmissions in >80% of pairs depending on analyses (Tables S2.3–S2.14, [30]).

**Table 1:** Infector probability results.

	Threshold 80%/90%		
Parameter/tree/perc.	Sensitivity^a^	Specificity^a^	Precision^a^
Group 1/true trees/5	0.02/0	1/1	0.8/NA
Group 1/1000 bp/5	0.04/0.006	1/1	0.78/1
Group 1/10 000 bp/5	0.03/0	1/1	0.83/NA
Group 1/true trees/60	0.024/0.006	0.997/0.99	0.78/0.81
Group 1/1000 bp/60	0.034/0.011	0.996/0.99	0.75/0.81
Group 1/10 000 bp/60	0.026/0.006	0.997/0.99	0.78/0.84
Group 1/true trees/90	0.026/0.007	0.996/0.99	0.82/0.89
Group 1/1000 bp/90	0.036/0.011	0.994/0.99	0.77/0.80
Group 1/10 000 bp/90	0.028/0.007	0.996/0.99	0.82/0.86
Group 2/true trees/5	0.005/0.005	1/1	0.67/1
Group 2/1000 bp/5	0.006/0.006	1/1	0.67/1
Group 2/10 000 bp/5	0.008/0.005	1/1	0.75/1
Group 2/true trees/60	0.014/0.004	0.998/0.99	0.78/0.81
Group 2/1000 bp/60	0.022/0.007	0.997/0.99	0.78/0.84
Group 2/10 000 bp/60	0.016/0.004	0.998/1	0.81/0.84
Group 2/true trees/90	0.018/0·005	0.997/1	0.86/0.90
Group 2/1000 bp/90	0.026/0·009	0.997/0.99	0.83/0.86
Group 2/10 000 bp/90	0.019/0·005	0.997/0.99	0.85/0.89

Sensitivity, specificity and precision for infector probabilities thresholds of 80% and 90% for true trees and ML trees estimated with 1000 bp and 10 000 bp for different sampling depth (percentage) of diagnosed individuals not on ART of 5%, 60% and 90%. Here, we quantified whether a pair represented a transmission pair independent of who infected whom for parameter group 1 and parameter group 2 and a total of 500 migrants per year to and from *region*.

a Sensitivity = TP/(TP + FN); specificity = TN/(TN + FP); precision = TP/(TP + FP), where TP is the number of true positives; FP is the number of false positives; TN is the number of true negatives; and FN is the number of false negatives. NA, no TP was observed.

### Analysis of next-generation sequencing

To understand the accuracy of phyloscanner to identify transmission pairs we also used PRCs. PRCs showed that phyloscanner performed better than a random classifier (Fig. 3c). We observed very high values of precision compared to recall for both parameter group values (Fig. 3c) and these PRCs showed an AUC of 68% and 70% for parameter groups 1 and 2, respectively. Similar results were observed for the different migration rates (Figure S14, Supplemental Digital Content). Note that for phyloscanner analyses, we observed precision values above 0.50 for the smallest threshold values used to generate the PRC (Fig. 3c).

The threshold used with phyloscanner to identify whether a pair represented a transmission pair is described in Supplementary Material. Our results showed that sensitivity is low because the absolute number of FN is higher than the TP and that specificity is very high because the absolute number of TN is very high compared to FP (Tables S20–S21, Supplemental Digital Content). We also observed high values for precision which were similar for analyses carried out for parameter groups 1 and 2 (Table 2 and Table S21, Supplemental Digital Content).

**Table 2:** Phyloscanner results.

*W* ≥ 80%/*W* ≥ 1%			
Parameter	Sensitivity^a^	Specificity^a^	Precision^a^
1	0.06/0.19	0.99/0.96	0.86/0.82
2	0.07/0.21	0.99/0.95	0.90/0.80

Sensitivity, specificity and precision for phyloscanner analyses. Here, we quantified whether a pair represented a transmission pair independent of who infected whom filtering data by infector probability (*W*) ≥80% or 1% and a total of 500 migrants to and from *region* per year.

a Sensitivity = TP/(TP + FN); specificity = TN/(TN + FP); precision = TP/(TP + FP), where TP is the number of true positives; FP is the number of false positives; TN is the number of true negatives; and FN is the number of false negatives.

We observed a higher number of TP pairs (Tables S20, Supplemental Digital Content) for data filtered by *W* ≥1% than by *W* ≥80%. This was a consequence of analyzing an average of eight times more pairs than filtering the data by *W* ≥80%. Finally, an average of 43.5% of pairs were identified as FN for any set of parameter group values (Table S20, Supplemental Digital Content). Conversely, only 6.5% of pairs were identified as TP. Within those TP pairs, phyloscanner correctly identified direct transmissions on an average of 99% pairs (for any combination of parameter values and migration rate) for both analyses using *W* ≥80% and *W* ≥1%.

When comparing the pairs identified as FP to the network simulations, we observed an average of 93% and 84% of one intermediary ID for pairs filtered by *W* ≥80% and *W* ≥1%, respectively. An intermediary ID is an unsampled viral genetic sequence from an individual who was in the network transmission simulation but was not sampled in the 5%–90% population depth. When we considered the intermediary ID, the order of transmissions followed a transmission chain in the form of host.1→intermediary(s)→host.2, where host.1 and host.2 were the pair analyzed by phyloscanner.

In contrast, for pairs identified as TN, we observed an average of 79% and 65% of one intermediary ID for pairs filtered by *W*≥80% and *W*≥1%, respectively. When checking the order of transmissions, most pairs (average of 87% and 88.5% for pairs filtered by *W* ≥ 80% and *W* ≥ 1%, respectively) followed a pattern of intermediary→host.1 and intermediary(s)→host.2; or intermediary→host.1 and host.2→intermediary, where host.1 and host.2 were the pair analyzed by phyloscanner. The remainder of pairs followed a pattern of host.1→intermediary→host.2.

## Discussion

The ability to attribute a direct transmission event to an individual is the greatest potential risk of HIV ME. We found that consensus sequence data pose minimal risk for these concerns, as using these data to estimate infector probabilities as a classifier is inadequate for forensic purposes but may still be useful for epidemiological investigations and aggregated analysis. The AUC for each PRC showed low and very similar values for the true trees and ML trees (Table S7, Supplemental Digital Content). Also, as expected, the number of pairs identified as TP and FP decreased with a higher infector probability threshold value (Tables S2.3–S2.14, [30]). These results held true independent of the epidemic parameters and migration rates evaluated.

There are several reports, summarized in Abecasis *et al.*[31], showing the use of phylogenetic trees together with epidemiological data as evidence to support or refute direct transmissions. However, we showed that analysis of consensus sequences alone provides only weak evidence of transmission directness, as the presence of unsampled individuals who may act as intermediaries in a transmission chain or common source of infection cannot be ruled out [31,32].

Alternatively, a recent study analyzing 32 couples using whole-genome NGS and phyloscanner correctly identified the source of transmission in >93% of couples (a sample highly skewed towards true transmission pairs) with no incorrect results [6]. However, this study [6] was carried out on a cohort in which epidemiological data linking individuals were available and their results might not extend to other datasets. Other recent studies using phyloscanner also analyzed biased samples representing data from linked individuals [5,6] or the authors applied a strategy to analyze sequences that potentially represented phylogenetically close pairs [10]. In contrast, we used random samples to understand whether phyloscanner could infer direct transmissions or transmission pairs. Even though NGS generates a high amount of sequencing data, we classified as TP only a small percentage (*ca.* 6.5%) of the pairs analyzed. From those classified as TP, phyloscanner was able to attribute the source of transmission in 99% of the cases. When comparing to previously reported results [6], our estimates of direct transmission attribution was slightly higher, most likely because in our simulations we have a lower error attributable to sequencing technologies than when carrying out real data experiments.

Phyloscanner rarely incorrectly identified transmission pairs (FP in Table S20, Supplemental Digital Content), however since this is a potentially costly mis-inference, we separately characterized how these errors occurred. A close analysis of FP pairs showed that there were one or more unsampled intermediaries in a transmission chain in which transmission happened very quickly.

Given our simulated data results, in the right setting with sampling performed soon after seroconversion and using HIV whole-genomes NGS, inference about transmission directionality may be significantly strengthened. However, we still showed that if unsampled intermediaries are present between a pair analyzed by phyloscanner, attribution of direct transmissions or transmission pairs may be incorrect. Based on these future directions, it is also important to have in place safeguards to anonymize data, but also make sure results are not over-interpreted.

There were several limitations in our study. First, our network simulation did not include assortativity by age or risk group. However, for the purposes of our analysis, we do not think assortativity would increase our ability to identify who transmitted to whom at the individual level. Second, we were unable to exactly match simulated epidemic trajectories with reported incidence from San Diego. The network model used here is commonly used with egocentric data for parameterization of the model [12]. To increase the complexity of the network model, it is important that the parameter values are based on realistic empirical data if available [12]. When simulating the San Diego HIV epidemic, many parameters lacked informative priors or were missing for some years of the HIV surveillance data. For example, there was no prior information available on the average number of partners per person for any time for MSM in San Diego. Finally, because our analyses required significant computational resources, our simulations were carried out on best case scenarios, such as good quality sequence alignments and for the *pol* and whole-genomes. However, analyses of empirical sequencing data with information linking who infected whom, showed that results obtained with whole-genomes and *pol* gene performed better than the using the *gag* or *env* genes [6].

In summary, our results suggest that source attribution using infector probabilities with consensus sequence data combined with clinical markers of stage of infection will seldom infer transmission pairs and direct transmissions with high confidence. This conclusion holds for a large range of sampling depths and whole-genomes versus partial genes. Detecting transmission pairs with whole-genome NGS data was rare. A small proportion (5%–10%) of transmission pairs within a random sample could be identified with high confidence. Within those transmission pairs, we inferred who infected whom with high confidence. For research settings with strong epidemiologic linkage, such as the one reported by Zhang *et al.*[6], there is a nonnegligible chance of identifying who infected whom.

## Acknowledgements

This study was supported by NIH MH124590, AI106039, MH100974, and by the James B. Pendleton Charitable Trust. The full SESAME research team guided development of these analyses including: Jeremy Sugarman, Gail Geller, Janesse Brewer, John Bridges, Juli Bollinger, Travis Sanchez, Anne Schuster, and Leslie Meltzer. This research was carried out using resources provided by the Research Computing Service facilities of the Imperial College London; the OSG (Open Science Grid), which is supported by the National Science Foundation and the U.S. Department of Energy's Office of Science; and (3) the Extreme Science and Engineering Discovery Environment (XSEDE), which is supported by National Science Foundation grant number ACI-1548562.

### Conflicts of interest

There are no conflicts of interest.

## Supplementary Material
