# Supplementary material for: Assessing transmission attribution risk from simulated sequencing data in HIV molecular epidemiology
Source: AIDS. 2024 Mar 4;38(6):865–73. doi: 10.1097/QAD.0000000000003820 (PMC10994139; doi:10.1097/QAD.0000000000003820)
Supplement: Supplementary file 1 [file aids-38-865-s001.docx]

**Supplementary Material**

**Assessing transmission attribution risk from simulated sequencing data in HIV molecular epidemiology**

Fabrícia F. Nascimento; Ph.D.^1*^, Sanjay R. Mehta; MD, DTM&H,D(ABMM)^2^, Susan J. Little; MD^2^, and Erik M. Volz; Ph.D.^1*^

^1^MRC Centre for Global Infectious Disease Analysis and the Department of Infectious Disease Epidemiology, Imperial College London, London, UK

^2^Division of Infectious Diseases, University of California San Diego, San Diego, CA, USA

***Corresponding authors**

[Methods 3](#_Toc148091181)

[Model framework overview 3](#_Toc148091182)

[Mixing by origin and target statistics 3](#_Toc148091183)

[Duration of partnerships 3](#_Toc148091184)

[Modules of R package 3](#_Toc148091185)

[Module to initialize individuals in the network 4](#_Toc148091186)

[Module for departures 4](#_Toc148091187)

[Module for arrivals 4](#_Toc148091188)

[Module for Ageing 4](#_Toc148091189)

[Module for migration 5](#_Toc148091190)

[Module for disease progression 5](#_Toc148091191)

[Module for HIV diagnostic testing 5](#_Toc148091192)

[Module for antiretroviral therapy 5](#_Toc148091193)

[Module for HIV transmission 6](#_Toc148091194)

[Transmission Rate and Transmission Probability 6](#_Toc148091195)

[Parameter Values Used in the Agent-Based Simulations 7](#_Toc148091196)

[Model Calibration and Simulations 8](#_Toc148091197)

[Simulation and analysis of consensus sequences 8](#_Toc148091198)

[Sampling of individuals 8](#_Toc148091199)

[Estimation of phylogenetic trees 9](#_Toc148091200)

[Simulation of sequence alignments 9](#_Toc148091201)

[Estimation of maximum likelihood and time-scaled trees 9](#_Toc148091202)

[Identification of transmission pairs using infector probabilities 9](#_Toc148091203)

[Accuracy of estimated infector probabilities 9](#_Toc148091204)

[Simulation and analysis of next-generation sequencing (NGS) 10](#_Toc148091205)

[Sampling of individuals 10](#_Toc148091206)

[Estimation of phylogenetic trees 10](#_Toc148091207)

[Simulation of sequence alignments 10](#_Toc148091208)

[Simulation and mapping of Illumina reads 10](#_Toc148091209)

[Identification of transmission pairs using phyloscanner 11](#_Toc148091210)

[Accuracy of phyloscanner 11](#_Toc148091211)

[Results and Discussion 12](#_Toc148091212)

[Parameter values 12](#_Toc148091213)

[Analysis of consensus sequences 13](#_Toc148091214)

[Analysis of NGS with phyloscanner 33](#_Toc148091215)

[References 35](#_Toc148091216)

# Methods

## Model framework overview

We described a simple mathematical model for HIV transmission in MSM (men-who-have-sex-with-men) from San Diego, CA, USA. We used a network-based transmission model developed in the R package EpiModel (version 2.2.1) [1] which allowed the simulation of complex network-based mathematical models of infectious diseases. In agent-based simulations, individuals simulated in the network were uniquely identifiable and were tracked over time allowing us to know who infected whom, time of infection and any metadata associated to each individual.

Using EpiModel, we simulated dynamic networks based on partnership formation and dissolution processes using STERGM (Separable Temporal Exponential Family Random Graph Modelling) [1,2]. STERGM jointly models the dissolution and formation of a tie or relationship to estimate and simulate complete networks given a set of target statistics from empirical data [3]. We also used an approximation of the STERGM because it is less computational intensive than a full STERGM [2].

For our dynamic simulations we used an offset to preserve the mean degree (the average number of partners per person on any time) given fluctuations on population size because of arrivals and deaths of individuals in the network. This offset was based on the network-size-invariant parametrization of Krivitsky *et al*. [4] in which the mean degree is preserved with fluctuations on network size.

Our mathematical model was developed as an R package HIVepisim that was built based on EpiModelHIV [5]. The main differences between HIVepisim and EpiModelHIV was the implementation of disease progression and HIV transmissions. In general, the network-based mathematical model implemented in HIVepisim was simpler than those implemented in EpiModelHIV and we did not stratify the population by ethnic groups.

### Mixing by origin and target statistics

Our network was simulated as a single network composed by two populations. These two populations were determined by an attribute named “origin” assigned to each individual in the network that could take two values: *region* or *global*. *Region* was representative of the MSM population in San Diego, while *global* was representative of a bigger population that would allow to account for importation of lineages from a larger global reservoir. Individuals from *region* could not form a relationship or tie with individuals from the *global* population.

We used a simple formation model in which partner selection was random and the degree distribution (number of ongoing partners) was binomial within each population. We were unable to find a report of mean degree for MSM in San Diego. Because of that we used for both *region* and *global* a mean degree of 1.19 which was the mean degree observed for MSM in the USA [6].

For the formation model, we used the terms *edges* and *nodemix* to specify the expected total number of edges in the network and expected total number of edges by the attribute “origin”, respectively. Target statistics for the expected total number of edges (or ties) in the network was set to (1*.*19 × *n_pop*1*/*2) + (1*.*19 × *n_pop*2*/*2); where *n_pop*1 and *n_pop*2 were the initial number of nodes (or individuals) in the network for *region* and *global*, respectively. Using *nodemix*, we specified the target statistics for the expected number of ties between individuals in *global* to 1*.*19 × *n_pop*2*/*2 and ties between individuals from *region* and *global* was set to zero. The expected number of ties within individuals from *region* was left as a free parameter and was estimated within EpiModel [1].

We simulated a network with 80,000 initial nodes in which 20,000 nodes were the initial number of individuals in *region* and 60,000 nodes were the initial number of individuals in *global*.

### Duration of partnerships

For the dissolution model, we used a heterogeneous and geometrically distributed process with a unique parameter for each type of relationship at each time step. We set the duration of a partnership between individuals in *region* to 24.03 days based on the estimated median partnership duration reported for MSM in San Diego [7]. We used the same partnership duration of 24.03 days for individuals in *global*.

### Modules of R package

Below we described the modules we developed in our R package HIVepisim as well as the parameter values used in the agent-based simulations. The time steps of all simulations were in units of days, and we simulated a total of 14,976 days, which was equivalent to 41 years or from 1st January 1980 to 1st January 2021.

### Module to initialize individuals in the network

This module initialized all individuals in the network. We simulated a population composed of only MSM individuals. Initial infected individuals were initialized in the network in acute and early HIV stage of infection, not diagnosed and not on antiretroviral therapy (ART). All additional attributes of initial infected individuals were initialized as “not known”. Additional attributes of infected individuals were time that AIDS started; time of HIV diagnosis; and time ART started. Susceptible individuals were initialized with all attributes related to an infected individual as “not known”. All initial individuals in the network were assigned with 80% and 20% to a low risk group and high risk group, respectively [8].

### Module for departures

Individuals could depart the network because of natural mortality or disease related mortality.

Natural mortality rates for *region* were based on the crude mortality rates reported for males in San Diego from 1980 to 2016 as downloaded from the CDC Wonder website (wonder.cdc.gov). Crude mortality rates were reported per 100,000 population. We assumed that 6.7% of the male population were MSM, based on estimates for the MSM population in San Diego [9]. We converted crude mortality rates to a rate per individual per day. We used this crude mortality rate for both *region* and *global* populations. These natural mortality rates were also stratified by age groups as reported by the wonder website: 15–19; 20–24; 25–34; 35–44; 45–54; 55–64; 65–74; and 75–84 years old (Table S2.1 [10]). We used linear interpolation to obtain the departure rate per time step.

Only infected individuals at stage 4 or AIDS could depart the network because of disease related mortality. For AIDS related mortality we used the rate per day per individual of 1*/*(5*.*06 × 365) [11].

At each time step, departures were simulated using a binomial (Bernouli) distribution using the departure rate as probability.

### Module for arrivals

MSM individuals arrived at the population at the age of 18 years old. At each time step, the number of individuals who arrived in the network were simulated based on a binomial (Bernouli) distribution using the arrival rate parameter as probability.

Arrival rates for the MSM population in San Diego (or *region*) were based on the crude birth rates obtained in the San Diego County website (www.sandiego county.gov). Crude birth rates were reported for births of males and females relative to the total population in San Diego from 2000 to 2019. We assumed that 6.7% of the male population were MSM, based on estimates for the MSM population in San Diego [9]. We used linear interpolation to obtain the arrival rate per time step.

Arrival rate for the *global* population were based on the global birth rate obtained in the worldbank website (data.worldbank.org/indicator/SP.DYN.CBRT.IN?end=2019&start=1980&view=chart) from 1980 to 2019. We also arbitrarily assumed that 6.7% of the *global* male population were MSM. We converted all crude birth rate per year to a birth rate per individual per day that was used as arrival rate in our simulations. We used linear interpolation to obtain the arrival rate per time step.

We simulated a total of 14,976 days. In the first initial 1,325 simulated time steps, individuals stochastically arrived in the network. After that, the total number of individuals who arrived the network was fixed to the same value as the total number of individuals who departed the network. This was done to keep the population at equilibrium and to avoid a situation in which all infected individuals died before the end of the simulation.

All new individuals in the network began as susceptible with 80% and 20% chance of being assigned to a low-risk and high-risk group, respectively [8]. Individuals in the high-risk group had a ten-fold increase in transmission compared to individuals in the low-risk group [8].

### Module for Ageing

The ageing of all individuals was linear by time step for all active individuals. Older individuals had a higher probability of departing the network because of natural mortality.

### Module for migration

This module allowed individuals to migrate between two populations and was controlled by two different rates: 1) A rate controlling the migration of individuals from *region* to *global*, and 2) a rate controlling the migration from *global* to *region*.

We arbitrarily set the migration rates in each direction to an average of 500 migrants per year, which was equivalent to 1.37 migrants per day. We also did a sensitive analysis for this parameter to an average of 250 and 750 migrants per year, which was equivalent to 0.68 and 2.05 migrants per day, respectively.

At each time step, migration was simulated based on a binomial (Bernouli) distribution and using the migration rate as probability.

### Module for disease progression

We modelled disease progression following Cori *et al*. [11] with five stages.

- Stage 0 was equivalent to acute and early HIV stage of infection.
- Stage 1 was equivalent to CD4 *>* 500 cells / mm^3^.
- Stage 2 was equivalent to 350 cells / mm^3^ *<* CD4 ≤ 500 cells / mm^3^.
- Stage 3 was equivalent to 200 cells / mm^3^ *<* CD4 ≤ 350 cells / mm^3^ and.
- Stage 4 or AIDS was equivalent to CD4 ≤ 200 cells / mm^3^.

Individuals in stage 0 could progress to any other stage of HIV infection at a rate of 1*/*(0*.*5×365) per day. After this short-lived acute and early HIV infection, an individual had a probability of 0.76, 0.19, 0.05 and 0 to progress to stages 1, 2, 3, and 4, respectively. Once in a chronic stage of HIV infection (represented by stages 1 to 3), an individual could progress from stage 1 to stage 2 at a rate of 1*/*(3.32 × 365) per day; from stage 2 to stage 3 at a rate of 1*/*(2.7 × 365) per day; and from stage 3 to stage 4 at a rate of 1*/*(5.5 × 365) per day. Only individuals not on ART could progress to another HIV stage of infection.

At each time step, disease progression was simulated based on a binomial (Bernouli) distribution using the different rates of disease progression as probability.

### Module for HIV diagnostic testing

The probability per day an MSM would be tested were estimated based on the published report on “HIV Trends and the Status of High Risk Groups in San Diego County, 1985–2001” obtained from the San Diego County website

(www.sandiegocounty.gov/content/sdc/hhsa/programs/phs/hiv_aids_epidemiology_unit/archives.html). We used the tables on Anonymous HIV Counselling and Testing Services for the number of MSM and males tested from 1990 to 2001. Because data were not available for earlier years, we made a best guess by estimating the probability an MSM would be tested in 1989 based on the total testers for that year and the average proportion of MSM of 0.22 which was obtained from 1990 to 2001. We used linear interpolation to obtain the probability an MSM would be tested per time step. See Table S2.2 [10] for values used in our simulations.

At each time step, we simulated HIV diagnostic testing using a binomial (Bernouli) distribution.

### Module for antiretroviral therapy

In our simulations, ART started in 1995. Only individuals that were diagnosed as HIV positive could receive treatment. Once an individual started treatment, they would stay on treatment. Individuals on treatment did not progress to another HIV stage of infection. We did not model HIV monotherapy before 1995; ART adherence (individuals who cycles on and off ART); and the appearance of drug-resistant virus. Because we lacked information on the probability and MSM would initiate treatment based on surveillance data, we sampled this parameter using a Latin hypercube sampling. For more details see section on Model Calibration and Simulations below.

At each time step, treatment was simulated based on a binomial (Bernouli) distribution.

### Module for HIV transmission

Transmission only happened between an HIV positive individual and an HIV negative individual and between individuals from same population or “origin”. We modelled transmissions following Le Vu *et al.* [8] and we assigned weights to the transmission probabilities by risk category, treatment status and stage of HIV infection.

We assigned a weight of 1 and 10 for low-risk group and high-risk group, respectively [8]. We assigned a weight of 1 for an individual not diagnosed with HIV; a weight of 0.5 for an individual diagnosed but not on ART; and a weight of 0.05 for an individual diagnosed and on ART [8]. Finally, we assigned a weight of 1 for individuals in stage 0 of HIV infection; a weight of 0.1 for individuals in stages 1 to 3; and a weight of 0.3 for an individual in stage 4 or AIDS [8].

Because this is a network model and relationships have a duration, the final transmission probability was also dependent on the act rate. In EpiModel, the act rate is defined as the number of transmissible acts per partnership per unit time.

### Transmission Rate and Transmission Probability

Estimates for the transmission rate per couple were not available for MSM in San Diego. To account for that we used equation 1 to estimate transmission rate (*β*) from 1980 to 2020 for MSM in San Diego.

*Incidence*(*t*) ≈ *β*(*t*)*I*(*t*) (1)

Incidence here is defined as the number of new infections per year, *β*(*t*) specifies the per-capita rate of transmissions from infected individuals and *I*(*t*) is the number of infected individuals.

We estimated HIV incidence for MSM in San Diego using the HIV Platform tool (www.ecdc.europa.eu/en/publications-data/hiv-platform-tool). The HIV Platform tool uses a multi-state back-calculation model to estimate HIV incidence using surveillance data [12].

We used aggregated surveillance data for HIV diagnosis from 1980 to 2020; AIDS diagnosis, simultaneous diagnosis and deaths from 1981 to 2020. We also used information on CD4 count from 2001 to 2020. CD4 count was separated into CD4 *<* 200 cells / mm^3^; 200 cells / mm^3^ *<* CD4 *<* 499 cells / mm^3^; 500 cells / mm^3^ *<* CD4 *<* 749 cells / mm^3^; and CD4 ≥ 750 cells / mm^3^. Aggregated HIV/AIDS surveillance information data was obtained by contacting the HIV Epidemiology Unit at the San Diego County (www.sandiegocounty.gov).

To run the model in the HIV Platform tool we also specified a four time intervals for changes in the diagnosis probabilities (a required parameter to run the HIV Platform tool). From 1980 to 1983; 1983 to 1995; and 1995 to 2020. For the last time interval, we allowed the probability of being diagnosed to change. We also tested many variations of time intervals for changes in the diagnosis probability, and no significant variation on the incidence curve were observed. Because of that we kept the simpler version as described here as it was easier and quicker to fit using the HIV Platform tool. See Figure S1 for the obtained incidence curve.

To convert the transmission rate *β*(*t*) to the transmission probability we used equation 2 [13].

*β*(*t*) = *m*[1 − (1 − *α*)*^n^*] (2)

where *m* is the average number of sex partners per year; *n* is the average number of intercourse acts per partner per year (or the act rate described in *Module for HIV transmission*); and *α* is the transmission probability.

To estimate the average number of sex partners per year (*m*), we assumed that the mean number of concurrent partners was a constant. In this case, the rate of partner formation (*r*) could be set to 1*/duration*. Duration of relationship for MSM in San Diego was set to 0.79 months [7]. We then set to *m* = (*mean degree* × 12*months*) */ duration*. *m* = (1*.*29 × 12) */* 0*.*79 = 19*.*59494. We arbitrarily set the act rate per day to 1, as this parameter was also not reported for MSM in San Diego. The value for act rate per year that we used to estimate the transmission rate was set to act rate in days × duration of relationship in days = 1 × 23.7 = 23*.*7 transmissible acts per partnership per year.

**Figure S1:** HIV incidence estimated using the HIV Platform tool.

### Parameter Values Used in the Agent-Based Simulations

In summary, the parameters used in our agent-based simulations described the 1) initial population size and structure; 2) migration in and out of San Diego (or *region*); 3) transmission probabilities; and 4) probability an MSM would be diagnosed and start ART over time (Table S1).

**Table S1: Parameter values:** List of parameter values used in the network transmission simulations

| **Parameter** | **Value** |
| --- | --- |
| *HIV stage progression rate*^a^ |  |
| From stage 0 to any other stage | 1*/*0*.*5*/*365 day^−1^ |
| From stage 1 to stage 2 | 1*/*3*.*32*/*365 day^−1^ |
| From stage 2 to stage 3 | 1*/*2*.*7*/*365 day^−1^ |
| From stage 3 to stage 4 (AIDS) | 1*/*5*.*5*/*365 day^−1^ |
| AIDS death | 1*/*5*.*06*/*365 day^−1^ |
| *Fraction of individuals transitioning from*^a^ |  |
| Stage 0 to stage 1 | 0.76 |
| Stage 0 to stage 2 | 0.19 |
| Stage 0 to stage 3 | 0.05 |
| Stage 0 to stage 4 | 0 |
| Proportion of individuals in low-risk group^b^ | 0.8 |
| Number of migrants to or from *region* | 500 per year |
| Initial population size in *region* | 20,000 individuals |
| Initial population size in *global* | 60,000 individuals |
| *Diagnosis probability* |  |
| Before 1985 | 0 |
| From 1985 | Varied through time^c^ |
| Probability an MSM will initiate treatment |  |
| Before 1995 | 0 |
| From 1995 | Varied through time^c^ |
| Transmission probability | ≈6*.*62×10^7 d^ |
| *Transmission weight conferred to individuals in*^b^ |  |
| Stage 0 | 1 |
| Stages 1 to 3 | 0.1 |
| Stage 4 | 0.3 |
| Care status as undiagnosed | 1 |
| Care status as diagnosed and not on ART | 0.5 |
| Care status as diagnosed and on ART | 0.05 |
| Low-risk group | 1 |
| High-risk group | 10 |
| Natural mortality rate | Varied through time^c^ |
| Arrival rate | Varied through time^c^ |

^a^HIV stages of infection were based in Cori *et al.* [11]. Stage 0 was equivalent to acute and early HIV stage of infection; stage 1 was equivalent to CD4 *>* 500 cells/mm^3^; stage 2 was equivalent to 350 *<* CD4 ≤ 500 cells/mm^3^; stage 3 was equivalent to 200 *<* CD4 ≤ 350 cells/mm^3^; and stage 4 or AIDS was equivalent to CD4 ≤ 200 cells/mm^3^.

^b^Based on Le Vu *et al.*[8]

^c^See methods described in this Supplementary Material.

^d^This parameter in the R package EpiModel is the probability of infection per transmissible act between a susceptible and an infected person.

## Model Calibration and Simulations

We used random Latin hypercube sampling (LHS) [14] design to sample six parameter values from a uniform distribution (Table S2) using the function randomLHS from the R package lhs version 1.1.3 [15]. For an explanation on how random LHS works, see their website at https://www.rdocumentation.org/packages/lhs/versions/0.14/topics/randomLHS.

The six parameter values we sampled were (1) the initial population size of HIV infected individuals in *region* (init_pop1_param); (2) the probability per day an MSM would initiate treatment (tx.init.prob.param); and four different scalars (3) scalar1; (4) scalar2; (5) scalar3 and (6) scalar4. Scalars 1 to 3 were used to increase/decrease the transmission probability parameter aiming at matching the incidence curve obtained with the HIV platform tool (Figure S1), while scalar4 was used to increase the model parameter determining the probability an MSM would be tested per day. We used a LHS because these parameter values were not available from the San Diego MSM surveillance data.

We ran one replicate for each combination of parameter value in the LHS and used trajectory matching to select the best fit simulated trajectory for incidence of diagnosis when compared to surveillance data for San Diego. For trajectory matching, we used a Poisson loss function for simulated and observed annual HIV diagnoses. We then resampled the best simulated trajectory.

We initially sampled 10,000 parameter values and used trajectory matching to choose the best simulated trajectory for the incidence of diagnosis available for San Diego. Based on these 10,000 combinations of parameter values, we narrowed down the values from the uniform distribution to sample additional 5,000 parameter values using LHS (Table S2). We also used trajectory matching to select the best fit simulated trajectory for the incidence of diagnosis available for San Diego.

The surveillance data for incidence of HIV diagnosis were obtained by contacting the HIV Epidemiology Unit at the San Diego County (www.sandiegocounty.gov).

**Table S2:** List of parameters sampled from a uniform distribution (U).

| **Parameter definition** | **Values1^a^** | **Values2^b^** |
| --- | --- | --- |
| Initial number of HIV infected individuals | U(10, 300) | U(40, 300) |
| Probability per day an MSM would initiate ART | U(0.0001, 0.01253765) | U(0.0001, 0.01253765) |
| Scalar1 | U(3000, 6000) | U(3000, 3500) |
| Scalar2 | U(3000, 7000) | U(4000, 5300) |
| Scalar3 | U(1500, 4000) | U(3500, 5000) |
| Scalar4 | U(1, 3) | U(1, 2) |

^a^ 10,000 initial combination of parameter values were sampled from these uniform distributions.

^b^ 5,000 combination of parameter values were sampled after narrowing down values based on the 10,000 initial parameter values.

## Simulation and analysis of consensus sequences

### Sampling of individuals

To sample individuals simulated in the networks, we carried out two different sampling strategies.

- For sampling strategy 1, we sampled individuals who were active (have not departed the network), diagnosed and not on ART at the time of sampling.
- For sampling strategy 2, we sampled individuals who were active and diagnosed, independent of ART-status at the time of sampling.

We randomly sampled a date in the past 15 years (time of sampling) and randomly sampled individuals in *region* by varying the sampling depth from 5%, 10%, 20%, 30%, 40%, 50%, 60%, 70%, 80% and 90%. These sampling depths are related to the number of infected individuals sampled from the network simulations. We randomly sampled individuals who acquired infection in *region*, and after sampling, we removed all individuals that were infected in *region*, but had migrated to *global* at or before time of sampling.

We also randomly sampled a date from beginning (1980-01-01) to end (2021-01-01) of network simulations and randomly sampled 2% of individuals who acquired infection in *global* to calibrate the molecular clock and to account for importation of lineages in our phylogenetic trees.

### Estimation of phylogenetic trees

We used the sampled individuals and dates together with the transmission matrix to obtain a phylogenetic tree that reflected between- and within-host viral evolution using the program VirusTreeSimulator (github.com/PangeaHIV/VirusTreeSimulator). To run VirusTreeSimulator, we used the values described in Ratmann *et al.* [16] that described a model of within-host evolution with parameter values that utilized a logistic effective population size function with parameters N_0_ (effective population size at time of infection) equal to 0.00593; growth rate equal to 2.851904 years and t50 (time point relative to the time of infection in backwards time, at which the population is equal to half its final asymptotic value, in the logistic model) equal to -2 years [17]. For each initial infected individual in the network, we attributed the source of infection to not available (NA) and the time of transmission to 1980. For all other transmission pairs, we used the time in days converted to years obtained during the network simulation. We merged all individual phylogenetic trees generated by VirusTreeSimulator into a single phylogenetic tree and referred to these trees as the *true trees*.

### Simulation of sequence alignments

We used the program Seq-Gen version 1.3.4 [18] to simulate the genetic sequence alignments of 1,000bp and 10,000bp as consensus sequences (similar to Sanger sequencing) as FASTA format. We used the HKY (Hasegawa-Kishino-Yano) nucleotide substitution model [19] with a transition transversion rate of 8.75 and a mean substitution rate of 0.0028 per site per year which were obtained for HIV phylogenetic trees [20]. Seq-Gen simulate sequence alignments that are free from gaps and ambiguous nucleotides.

### Estimation of maximum likelihood and time-scaled trees

We used the simulated genetic sequence alignments to estimate phylogenetic trees using maximum likelihood (ML) using the program IQ-TREE 2 version 2.2.0 [21] and the HKY nucleotide substitution model [19,22]. We used the option -czb to collapse branch lengths with values near to zero, so as the final tree could be multifurcating.

We then estimated time-scaled trees using the ML trees and the uncorrelated lognormal relaxed clock model from the R package treedater version 0.5.3 [23]. We used the time of sampling described for the true trees and considered the alignment lengths of 1,000bp or 10,000bp. Note that the ML trees do not need to be rooted as *treedater* uses an algorithm to root the trees.

### Identification of transmission pairs using infector probabilities

To calculate infector probabilities, we used the function phylo.source.attribution.hiv.msm of the R package phydynR version 0.2.0 [24]. This function required a time-scaled phylogenetic tree, sampling times, CD4 count which was estimated based on the HIV stage of infection (as we only simulated HIV stages of infection) and identification of individuals in acute and early HIV stage of infection in the past 6 months. We supplied the average of the total number of individuals living with HIV and the number of new infections in the last simulated year using the results of the network simulations. To estimate infector probabilities, we used a time-scaled tree in which we removed sequences from *global* and kept only sequences from *region*.

### Accuracy of estimated infector probabilities

To evaluate the accuracy of estimated infector probabilities obtained with consensus sequences, we used precision-recall curves (PRC). To calculate a PRC, we used thresholds of 0 to 1 by increments of 0.01. For each threshold value, we categorize an infector probability above the threshold as a direct transmission pair irrespective of direction (i.e., A infected B or B infected A without intermediary individuals), while an infector probability below the threshold is categorized as not a transmission pair. We then compared these predicted values with observed values and calculated the *recall* (same as true positive rate or sensitivity) and *precision* (same as positive predictive value). Recall is defined as *TP /* (*TP* + *FN*) and is our ability to detect a true positive (TP) or a true transmission pair among all pairs that represented a true transmission pair while precision is defined as *TP /* (*TP* + *FP*) and is our ability to correctly predict a TP or transmission pair among all pairs predicted as a transmission pair.

Note that TP is the number of true positives or true transmission pairs; FN is the number of false negatives (i.e., pairs that were classified as not a transmission pair, but the observed data showed that they were in fact a transmission pair) and FP is the number of false positives (i.e., pairs that were classified as a transmission pair but the observed data showed that they were not a transmission pair). We were able to estimate TP, FP, TN, and FN because we know the observed values of who infected whom based on the network simulations.

## Simulation and analysis of next-generation sequencing (NGS)

### Sampling of individuals

We randomly sampled a date in the past five years (time of sampling) and randomly sampled approximately 90% of diagnosed individuals not on ART. We randomly sampled individuals who acquired infection in *region*, and after sampling, we removed the individuals who migrated to *global* at or before time of sampling.

### Estimation of phylogenetic trees

We used the sampled individuals and dates together with the transmission matrix to obtain a phylogenetic tree that reflected between- and within-host viral evolution using the program VirusTreeSimulator and used the same parameter values as described in the section for consensus sequences. The difference is that we simulated 10 proviruses for each individual to mimic viral diversity. The choice of 10 proviruses was based on a file size of Illumina reads from real data (provided by Dr. A. Chaillon).

We randomly removed all but one sequence per individual from the phylogenetic tree obtained with VirusTreeSimulator to obtain a consensus tree (a tree composed of only one sequence per individual). We used this consensus tree to estimate infector probabilities (*W*) and select two different dataset, one dataset containing all pairs that showed *W* ≥ 80% and a second dataset containing all pairs that showed *W* ≥ 1%. Note that infector probabilities are estimated on phylogenetic trees composed of only one viral sequence (or consensus sequence) per individual.

### Simulation of sequence alignments

We also used the program Seq-Gen [18] to simulate genetic sequence alignments (free of gaps and ambiguous nucleotides) that was used as input data to simulate synthetic Illumina reads (see sections below) and used the same parameter values as described for the analysis of consensus sequences. We provided a HIV-1 subtype B sequence (GenBank accession number: K03455) for Seq-Gen to use it as ancestral sequence at the root.

Here we simulate one sequence alignment per phylogenetic tree as FASTA format. We then split each sequence alignment into smaller alignments composed of 10 sequences per individual. Each FASTA alignment composed of 10 sequences per individual was used with ART_Illumina for the simulation of Illumina reads (see below). ART_Illumina will generate synthetic Illumina reads as FASTQ format that will be used as output for the simulation and mapping of Illumina reads (see next section).

### Simulation and mapping of Illumina reads

We used the program ART_Illumina version 2.5.8 [25] to simulate 250bp Illumina paired-end reads. We used the MiSeq v3 option to simulate 10,000 times coverage with a minimum and maximum base quality of 25 and 40, respectively. See Table S3 for list of parameters used with ART_Illumina to simulate reads.

We used the program SHIVER to map the Illumina reads with default values but set to false the option to trim for primers and adapters because our simulated Illumina paired-end reads did not have any primers or adapters attached to them. To initialize SHIVER, we used the 2020 reference genome alignment consisting of 42 sequences that we downloaded from the LANL (Los Alamos National Laboratory) HIV database (www.hiv.lanl.gov/content/sequence/NEWALIGN/align.html). We mapped Illumina reads to a reference subtype B instead of carrying out *de novo* assembly of the reads prior to SHIVER to reduce computation time.

By default, ART_Illumina selects a built-in quality score profile according to the read length specified for the run.

**Table S3:** Parameter values used to simulate Illumina reads.

| **Parameter** | **Description** | **Value** |
| --- | --- | --- |
| -ss | The name of Illumina sequencing system of the built-in profile used for simulation | MSv3 |
| -i | The filename of input DNA / RNA reference |  |
| -o | The prefix of output filename |  |
| -p | Indicate a paired-end read simulation |  |
| -l | The length of reads to be simulated | 250 |
| -m | The mean size of DNA / RNA fragments for paired-end simulations | 600 |
| -s | The standard deviation of DNA / RNA fragment size for paired-end simulations | 400 |
| -f | The fold of read coverage to be simulated | 10000 |
| -qL | The minimum base quality score | 25 |
| -qU | The maximum base quality score | 40 |
| -na | Do not output ALN alignment file |  |
| -ir | The first-read insertion rate | default: 0.00009 |
| -ir2 | The second-read insertion rate | default: 0.00015 |
| -dr | The first-read deletion rate | default: 0.00011 |
| -dr2 | The second-read deletion rate | default: 0.00023 |
| -k | The maximum total number of insertion and deletion per read | default: up to read length |

### Identification of transmission pairs using phyloscanner

Two steps are required to run a phyloscanner analysis.

- In the first step, we created read alignments based on a window width of 340bp and window overlap of 170bp [26]. These read alignments were used with IQ-TREE to reconstruct ML phylogenetic trees. For parameter values used in the first step of phyloscanner analyses see Table S4.
- In the second step, we inferred whether two IDs represented a transmission pair based on ancestral host-state reconstructions revealed by the phylogenetic trees based on each window alignment. For parameter values used in the second step of phyloscanner analyses see Table S5.

There are no universal guidelines to determine whether a pair represent a transmission pair using phyloscanner. Here we used the same values as described by Zhang *et al*. [26]. A pair was classified as linked by phyloscanner if ≥ 50% of windows showed an ancestral or complex relationship with minimum subgraph distance of *<*0*.*05 substitutions per site. A pair involved in a direct transmission event was assumed to be correct if the pair of individuals was linked and if the correct ancestral relationship (partner 1 to partner 2) was observed in ≥ 37*.*5% of windows with a minimum subgraph distance of *<*0*.*05 substitutions per site. On the other hand, a pair involved in a direct transmission event was assumed to be incorrect if the pair of individuals was linked and the incorrect ancestral relationship (partner 2 to partner 1) was observed in ≥ 37*.*5% of windows with a minimum subgraph distance of *<*0*.*05 substitutions per site [26].

### Accuracy of phyloscanner

To evaluate the accuracy of phyloscanner to identify a transmission pair, independent of who infected whom, we compared the results obtained with phyloscanner and network simulations and quantified the total number of pairs identified as true positives (TP), false positives (FP), true negatives (TN) and false negatives (FN). TP was estimated as the subset of transmission pairs correctly identified by phyloscanner using the filtering by W ≥ 80% or W ≥ 1%; FP was estimated as the subset of non-transmission pairs incorrectly identified as a transmission pair by phyloscanner using the filtering by W ≥ 80% or W ≥ 1%; TN was estimated as the subset of all non-transmission pairs within the sampled pairs in the simulations and not identified as FP by phyloscanner; and FN was estimated as the subset of all transmission pairs within the sampled pairs in the simulations but not identified as TP by phyloscanner.

**Table S4: Part 1 of phyloscanner analysis:** List of parameters used to generate sequence alignments using the python script phyloscanner_make_trees.py.

| **Parameter** | **Value** | **Description** |
| --- | --- | --- |
| Input file | csv file | Input file as csv format containing the location of the bam and reference files. |
| windows | Start and end coordinates of genomic windows from 800 to 9400 in 170bp increments in 340bp windows | We used values as described Zhang *et al*. [26] as this achieved best results with real dataset. |
| alignment-of-other-refs | Alignments of reference sequences | Subtype B GenBank accession number of K03455 and Subtype C GenBank accession number of AF443088 |
| pairwise-align-to | Subtype B K03455 | Name of the reference sequence against which to map genomic coordinates |
| merge-paired-reads | Flag was set | Option was used because we simulated paired-end Illumina reads. |
| quality-trim-ends | 25 | Threshold to trim ends of Illumina reads |
| min-internal-quality | 25 | Illumina reads with 2 or more based below this quality threshold were discarded. |
| excision-ref | Subtype B K03455 | This option was used to specify a reference in which the coordinates were specified with the option pairwise-align-to. |
| excision-coords | DrugResistancePositionsInHXB2.txt  (Available with phyloscanner) | Used to remove drug resistance positions |
| merging-threshold-a | 1 | Genetic similarity threshold to merge similar Illumina reads. |
| min-read-count | 2 | This option was used to specify a minimum count for each unique Illumina read. |
| no-trees | Flag was set | We used this flag for phyloscanner to generate only read alignments. We estimated phylogenetic trees using IQ-TREE. |

**Table S5:** **Part 2 of phyloscanner analysis:** List of parameters used to analyze phylogenetic trees using the R script phyloscanner_analyse_trees.R

x

| **Parameter** | **Value** | **Description** |
| --- | --- | --- |
| splitsRule | S | Sankoff algorithm |
| kParam | 20 | K parameters in Sankoff cost matrix. |
| outgroupName | Subtype C AF443088 | Reference sequences used as outgroup. |
| multifurcationThreshold | g | Threshold to collapse branches into polytomies. The “g” value means that threshold value will be guessed based on the trees itself. |
| allowMultiTrans | Flag was set | Using this option allows directionality between two individuals to be inferred when one or both individuals had more than one subgraph, and all subgraphs of one individual were ancestral to all subgraphs of the other individual. |

# Results and Discussion

## Parameter values

Table S6 shows the two best combination of parameter values for the simulation of network transmissions using trajectory matching as described in Methods.

Note that we had to sample from a random LHS six parameter values for the network simulations. After using trajectory matching, two best parameter group values, as defined in Table S6, were selected, and used in all further analysis to simulate 30 replicates of network transmissions using parameter group 1 and 30 replicates of network transmissions using parameter group 2 (for values see Table S6).

**Table S6:** **Parameter values:** The two best parameter group values, here referred to as parameter group 1 and parameter group 2, obtained with trajectory matching.

| **Parameter definition** | **Parameter**  **group 1** | **Parameter**  **group 2** |
| --- | --- | --- |
| Probability an MSM would initiate treatment | 0.00045 | 0.00031 |
| Initial number of infected individuals in *region* | 43 | 182 |
| scalar1^*^ | 3031.983 | 3190.817 |
| scalar2^*^ | 4295.164 | 4139.738 |
| scalar3^*^ | 4397.545 | 4008.211 |
| scalar4^*^ | 1.98 | 1.82 |

^*^ Scalars 1 to 3 was a number used to increase/decrease the transmission probability parameter in the network simulations, while scalar4 was a number used to increase the model parameter determining the probability an MSM would be tested per day.

## Analysis of consensus sequences

To evaluate the performance of infector probabilities in predicting true transmission pairs, we calculated precision-recall curves (PRC, see Methods). A PRC is represented by a line showing P(t)/(P(t)+N(t)) where P is the observed absolute number of positives or true transmission pairs while N is the observed absolute number of negatives or non-transmission pairs and *t* is the threshold infector probability used for categorizing transmission pairs. For a complete description of the theory of PRC on imbalanced data see Saito and Rehmsmeier [25].

Our results consistently showed that the performance of infector probabilities was better than a random classifier, represented by the horizontal lines in every plot. Results were similar independent of sampling strategy and analysis carried out with the true trees or ML trees estimated with 1,000bp or 10,000bp or migration rates (Figures S2 to S13).

**Figure S2:** **Precision-recall curves for sampling strategy 1, average of 250 migrants per year and parameter group 1.** Plot showing the precision-recall curves (PRC) for different sampling depth (5% – 90%) of the diagnosed individuals not on ART for adjacent tips in the true trees and ML trees estimated with 1,000bp and 10,000bp for an average of 250 migrants per year to and from *region*.

**Figure S3:** **Precision-recall curves for sampling strategy 1, average of 250 migrants per year and parameter group 2.** Plot showing the precision-recall curves (PRC) for different sampling depth (5% – 90%) of the diagnosed individuals not on ART for adjacent tips in the true trees and ML trees estimated with 1,000bp and 10,000bp for an average of 250 migrants per year to and from region.

**Figure S4:** **Precision-recall curves for sampling strategy 1, average of 500 migrants per year and parameter group 1.** Plot showing the precision-recall curves (PRC) for different sampling depth (5% – 90%) of the diagnosed individuals not on ART for adjacent tips in the true trees and ML trees estimated with 1,000bp and 10,000bp for an average of 500 migrants per year to and from *region*.

**Figure S5:** **Precision-recall curves for sampling strategy 1, average of 500 migrants per year and parameter group 2.** Plot showing the precision-recall curves (PRC) for different sampling depth (5% – 90%) of the diagnosed individuals not on ART for adjacent tips in the true trees and ML trees estimated with 1,000bp and 10,000bp for an average of 500 migrants per year to and from *region*.

**Figure S6:** **Precision-recall curves for sampling strategy 1, average of 750 migrants per year and parameter group 1.** Plot showing the precision-recall curves (PRC) for different sampling depth (5% – 90%) of the diagnosed individuals not on ART for adjacent tips in the true trees and ML trees estimated with 1,000bp and 10,000bp for an average of 750 migrants per year to and from *region*.

**Figure S7:** **Precision-recall curves for sampling strategy 1, average of 750 migrants per year and parameter group 2.** Plot showing the precision-recall curves (PRC) for different sampling depth (5% – 90%) of the diagnosed individuals not on ART for adjacent tips in the true trees and ML trees estimated with 1,000bp and 10,000bp for an average of 750 migrants per year to and from region.

**Figure S8: Precision-recall curves for sampling strategy 2, average of 250 migrants per year and parameter group 1.** Plot showing the precision-recall curves (PRC) for different sampling depth (5% – 90%) of the diagnosed individuals independent of ART-status for adjacent tips in the true trees and ML trees estimated with 1,000bp and 10,000bp for an average of 250 migrants per year to and from *region*.

**Figure S9: Precision-recall curves for sampling strategy 2, average of 250 migrants per year and parameter group 2.** Plot showing the precision-recall curves (PRC) for different sampling depth (5% – 90%) of the diagnosed individuals independent of ART-status for adjacent tips in the true trees and ML trees estimated with 1,000bp and 10,000bp for an average of 250 migrants per year to and from *region*.

**Figure S10:** **Precision-recall curves for sampling strategy 2, average of 500 migrants per year and parameter group 1.** Plot showing the precision-recall curves (PRC) for different sampling depth (5% – 90%) of the diagnosed individuals independent of ART-status for adjacent tips in the true trees and ML trees estimated with 1,000bp and 10,000bp for an average of 500 migrants per year to and from *region*.

**Figure S11:** **Precision-recall curves for sampling strategy 2, average of 500 migrants per year and parameter group 2.** Plot showing the precision-recall curves (PRC) for different sampling depth (5% – 90%) of the diagnosed individuals independent of ART-status for adjacent tips in the true trees and ML trees estimated with 1,000bp and 10,000bp for an average of 500 migrants per year to and from *region*.

**Figure S12:** **Precision-recall curves for sampling strategy 2, average of 750 migrants and parameter group 1.** Plot showing the precision-recall curves (PRC) for different sampling depth (5% – 90%) of the diagnosed individuals independent of ART-status for adjacent tips in the true trees and ML trees estimated with 1,000bp and 10,000bp for an average of 750 migrants per year to and from *region*.

**Figure S13:** **Precision-recall curves for sampling strategy 2, average of 750 migrants per year and parameter group 2.** Plot showing the precision-recall curves (PRC) for different sampling depth (5% – 90%) of the diagnosed individuals independent of ART-status for adjacent tips in the true trees and ML trees estimated with 1,000bp and 10,000bp for an average of 750 migrants per year to and from *region*.

The area under the curve (AUC) of PRC can be used as a score for comparison between PRCs. In general, the AUC for PRC constructed for sampling depth of 5% of diagnosed individuals were worse than the AUC for PRC constructed for a sampling depth of 90% (Table S7).

**Table S7:** **Area under the curve (AUC) for precision-recall curves for consensus sequences.** AUC values for sampling depth (perc.) of 5%, 60% and 90% of the diagnosed individuals not on ART and an average of 500 migrants per year to from *region* for parameter groups 1 and 2.

| **Parameter** | **Perc.** | **True trees** | **ML 1,000bp** | **ML 10,000bp** |
| --- | --- | --- | --- | --- |
| Group 1 | 5 | 20% | 24% | 22% |
| Group 1 | 60 | 31% | 31% | 31% |
| Group 1 | 90 | 37% | 36% | 37% |
| Group 2 | 5 | 11% | 12% | 11% |
| Group 2 | 60 | 32% | 33% | 32% |
| Group 2 | 90 | 39% | 39% | 40% |

Because there are no universal guidelines on which value of *W* should be used to indicate a transmission pair, we showed the absolute number of TP, FP, TN, and FN for *W* thresholds of 80% and 90% for comparisons for different sampling depths (ranging from 5% to 90%) of the diagnosed individuals not on ART for parameter groups 1 and 2 and different migration rates (Tables S2.3–S2.8 [10]).

For both thresholds and independent of migration rates and tree analyzed (true trees or ML trees), we observed that only a small percentage of pairs analyzed were identified as TP or true transmission pairs.

For a threshold of 80%, the percentage of pairs identified as TP varied from 0.1% to 1.3%, while for a threshold of 90%, it varied from 0 to 0.5%. On the other hand, for a threshold of 80%, the percentage of pairs identified as FP (i.e., it is predicted as a transmission pair when in fact it is not a transmission pair) varied from 0% to 0.4%, while for a threshold of 90%, it varied from 0% to 0.1%.

Similarly, we also showed the absolute number of TP, FP, TN, and FN for *W* thresholds of 80% and 90% and for different sampling depths (ranging from 5% to 90%) of the diagnosed individuals independent of ART-status for parameter groups 1 and 2 and different migration rates (Tables S2.9–S2.14 [10]).

For both thresholds and independent of migration rates and tree analyzed (true trees or ML trees), we observed that only a small number of pairs analyzed were identified as TP or true transmission pairs.

For a threshold of 80%, the percentage of pairs from the total pairs analyzed identified as TP varied from 0.1% to 1%, while for a threshold of 90%, it varied from 0 to 0.3%. On the other hand, for a threshold of 80%, the percentage of pairs identified as FP (i.e., it is predicted as a transmission pair when in fact it is not a transmission pair) varied from 0.02% to 0.2%, while for a threshold of 90%, it varied from 0% to 0.04%.

We showed that even though we could identify true transmission pairs using consensus sequences and infector probabilities, these represented a very small number/percentage of pairs analyzed. When using W>90% as indicative of a potential transmission pair, we showed the identification of a smaller number of pairs as TP and FP compared to using a W>80%.

When checking if we could attribute the source of a transmission event among TP pairs, we observed that for a population depth of 5%, we correctly identified direct transmissions in almost all pairs analyzed. However, this represented only very small number (less than 0.1%) of pairs from the total analyzed (Tables S2.3–S2.14 [10]). As we increased sampling depth, we identified direct transmissions in >80% of pairs depending on the tree analyzed. By using a threshold of 90%, we observed a smaller number of TP and FP compared to a threshold of 80% (Tables S2.3–S2.14 [10]). However, we increased the proportion of direct transmissions identified among those classified as TP.

Using the values of TP, FP, TN, and FN we derived sensitivity, specificity and precision for *W* thresholds of 80% and 80%. Sensitivity is defined as *TP /* (*TP* + *FN*) and is our ability to detect a true positive (TP) or a true transmission pair among all pairs that represented a true transmission pair. Specificity is defined as TN / (TN + FP) and is our ability to detect a true negative (TN) or a non-transmission pair among all pairs that represent a true non-transmission pair. Finally, precision is defined as *TP /* (*TP* + *FP*) and is our ability to correctly predict a TP or transmission pair among all pairs predicted as a transmission pair.

Results showed that specificity was very high which means that we could correctly identify non-transmission pairs. On the other hand, sensitivity was low showing that infector probabilities could identify few pairs as TP compared to the large number of pairs identified as FN. Finally, precision varied from approximately 30% to 80% depending on sampling strategy and sampling depth (Tables S8–S19). In general, a lower value of precision was observed for the smaller sampling depth of 5% and 10%.

**Table S8: Infector probability results for sampling strategy 1 for an average of 250 migrants per year to and from *region* for parameter group 1.** Sensitivity, specificity and precision for infector probabilities for two thresholds (80% and 90%) for true trees and ML trees estimated with 1,000bp and 10,000bp for different sampling depth of 5 to 90% (perc.) of diagnosed individuals not on ART. Here we quantified whether a pair represented a transmission pair independent of who infected whom for combination of parameters 1.

| **Parameter / Tree / Perc.** | **Sensitivity** | **Specificity** | **Precision** |
| --- | --- | --- | --- |
| Group 1 / True trees / 5 | 0.012 / 0 | 0.999 / 1 | 0.375 / 0 |
| Group 1 / 1,000bp / 5 | 0.014 / 0 | 0.999 / 1 | 0.333 / 0 |
| Group 1 / 10,000bp / 5 | 0.013 / 0 | 0.999 / 1 | 0.375 / 0 |
| Group 1 / True trees / 10 | 0.015 / 0.004 | 0.999 / 1 | 0.737 / 0.8 |
| Group 1 / 1,000bp / 10 | 0.014 / 0.005 | 1 / 1 | 0.733 / 0.8 |
| Group 1 / 10,000bp / 10 | 0.016 / 0.004 | 1 / 1 | 0.824 / 0.8 |
| Group 1 / True trees / 20 | 0.019 / 0.005 | 0.998 / 1 | 0.671 / 0.765 |
| Group 1 / 1,000bp / 20 | 0.028 / 0.01 | 0.997 / 1 | 0.583 / 0.75 |
| Group 1 / 10,000bp / 20 | 0.02 / 0.006 | 0.998 / 1 | 0.659 / 0.833 |
| Group 1 / True trees / 30 | 0.016 / 0.004 | 0.998 / 0.99 | 0.648 / 0.634 |
| Group 1 / 1,000bp / 30 | 0.023 / 0.007 | 0.997 / 0.999 | 0.605 / 0.717 |
| Group 1 / 10,000bp / 30 | 0.017 / 0.005 | 0.998 / 0.999 | 0.651 / 0.674 |
| Group 1 / True trees / 40 | 0.021 / 0.006 | 0.997 / 0.999 | 0.703 / 0.789 |
| Group 1 / 1,000bp / 40 | 0.029 / 0.009 | 0.996 / 0.999 | 0.678 / 0.74 |
| Group 1 / 10,000bp / 40 | 0.022 / 0.006 | 0.997 / 0.999 | 0.715 / 0.781 |
| Group 1 / True trees / 50 | 0.024 / 0.007 | 0.996 / 0.999 | 0.747 / 0.808 |
| Group 1 / 1,000bp / 50 | 0.033 / 0.011 | 0.995 / 0.999 | 0.706 / 0.78 |
| Group 1 / 10,000bp / 50 | 0.025 / 0.008 | 0.996 / 0.999 | 0.734 / 0.822 |
| Group 1 / True trees / 60 | 0.024 / 0.006 | 0.996 / 0.999 | 0.77 / 0.81 |
| Group 1 / 1,000bp / 60 | 0.032 / 0.009 | 0.995 / 0.999 | 0.746 / 0.772 |
| Group 1 / 10,000bp / 60 | 0.025 / 0.006 | 0.996 / 0.999 | 0.764 / 0.783 |
| Group 1 / True trees / 70 | 0.026 / 0.007 | 0.996 / 0.999 | 0.78 / 0.832 |
| Group 1 / 1,000bp / 70 | 0.035 / 0.011 | 0.995 / 0.999 | 0.753 / 0.787 |
| Group 1 / 10,000bp / 70 | 0.027 / 0.007 | 0.995 / 0.999 | 0.774 / 0.808 |
| Group 1 / True trees / 80 | 0.027 / 0.007 | 0.995 / 0.999 | 0.781 / 0.833 |
| Group 1 / 1,000bp / 80 | 0.038 / 0.012 | 0.994 / 0.998 | 0.764 / 0.799 |
| Group 1 / 10,000bp / 80 | 0.028 / 0.007 | 0.995 / 0.999 | 0.777 / 0.813 |
| Group 1 / True trees / 90 | 0.028 / 0.007 | 0.995 / 0.999 | 0.811 / 0.857 |
| Group 1 / 1,000bp / 90 | 0.037 / 0.012 | 0.993 / 0.999 | 0.772 / 0.834 |
| Group 1 / 10,000bp / 90 | 0.03 / 0.008 | 0.995 / 0.999 | 0.81 / 0.873 |

**Table S9: Infector probability results for sampling strategy 1 for an average of 250 migrants per year to and from *region* for parameter group 2.** Sensitivity, specificity and precision for infector probabilities for two thresholds (80% and 90%) for true trees and ML trees estimated with 1,000bp and 10,000bp for different sampling depth of 5 to 90% (perc.) of diagnosed individuals not on ART. Here we quantified whether a pair represented a transmission pair independent of who infected whom for combination of parameters 2.

| **Parameter / Tree / Perc.** | **Sensitivity** | **Specificity** | **Precision** |
| --- | --- | --- | --- |
| Group 2 / True trees / 5 | 0.017 / 0.004 | 1 / 1 | 0.75 / 1 |
| Group 2 / 1,000bp / 5 | 0.017 / 0.006 | 1 / 1 | 0.8 / 1 |
| Group 2 / 10,000bp / 5 | 0.016 / 0.007 | 1 / 1 | 0.818 / 0.8 |
| Group 2 / True trees / 10 | 0.008 / 0.001 | 0.999 / 1 | 0.625 / 0.5 |
| Group 2 / 1,000bp / 10 | 0.01 / 0.004 | 0.999 / 1 | 0.517 / 1 |
| Group 2 / 10,000bp / 10 | 0.011 / 0.003 | 0.999 / 1 | 0.645 / 0.75 |
| Group 2 / True trees / 20 | 0.012 / 0.003 | 0.999 / 1 | 0.727 / 0.742 |
| Group 2 / 1,000bp / 20 | 0.017 / 0.005 | 0.999 / 1 | 0.786 / 0.839 |
| Group 2 / 10,000bp / 20 | 0.013 / 0.004 | 0.999 / 1 | 0.739 / 0.852 |
| Group 2 / True trees / 30 | 0.01 / 0.002 | 0.999 / 1 | 0.705 / 0.853 |
| Group 2 / 1,000bp / 30 | 0.015 / 0.005 | 0.998 / 1 | 0.661 / 0.864 |
| Group 2 / 10,000bp / 30 | 0.011 / 0.003 | 0.998 / 1 | 0.68 / 0.814 |
| Group 2 / True trees / 40 | 0.013 / 0.004 | 0.998 / 1 | 0.773 / 0.859 |
| Group 2 / 1,000bp / 40 | 0.02 / 0.006 | 0.998 / 0.999 | 0.77 / 0.781 |
| Group 2 / 10,000bp / 40 | 0.015 / 0.004 | 0.998 / 1 | 0.766 / 0.88 |
| Group 2 / True trees / 50 | 0.015 / 0.005 | 0.998 / 1 | 0.809 / 0.867 |
| Group 2 / 1,000bp / 50 | 0.022 / 0.007 | 0.997 / 0.999 | 0.779 / 0.834 |
| Group 2 / 10,000bp / 50 | 0.016 / 0.005 | 0.998 / 1 | 0.805 / 0.905 |
| Group 2 / True trees / 60 | 0.016 / 0.004 | 0.997 / 0.999 | 0.802 / 0.855 |
| Group 2 / 1,000bp / 60 | 0.023 / 0.007 | 0.997 / 0.999 | 0.783 / 0.804 |
| Group 2 / 10,000bp / 60 | 0.017 / 0.005 | 0.997 / 0.999 | 0.802 / 0.844 |
| Group 2 / True trees / 70 | 0.016 / 0.005 | 0.997 / 1 | 0.815 / 0.896 |
| Group 2 / 1,000bp / 70 | 0.025 / 0.008 | 0.996 / 0.999 | 0.793 / 0.853 |
| Group 2 / 10,000bp / 70 | 0.018 / 0.005 | 0.997 / 0.999 | 0.813 / 0.885 |
| Group 2 / True trees / 80 | 0.018 / 0.005 | 0.996 / 0.999 | 0.827 / 0.899 |
| Group 2 / 1,000bp / 80 | 0.026 / 0.008 | 0.996 / 0.999 | 0.794 / 0.829 |
| Group 2 / 10,000bp / 80 | 0.019 / 0.005 | 0.997 / 0.999 | 0.847 / 0.861 |
| Group 2 / True trees / 90 | 0.021 / 0.006 | 0.996 / 0.999 | 0.854 / 0.881 |
| Group 2 / 1,000bp / 90 | 0.029 / 0.01 | 0.996 / 0.999 | 0.826 / 0.879 |
| Group 2 / 10,000bp / 90 | 0.022 / 0.006 | 0.996 / 0.999 | 0.852 / 0.899 |

**Table S10: Infector probability results for sampling strategy 1 for an average of 500 migrants per year to and from *region* for parameter group 1.** Sensitivity, specificity and precision for infector probabilities for two thresholds (80% and 90%) for true trees and ML trees estimated with 1,000bp and 10,000bp for different sampling depth of 5 to 90% (perc.) of diagnosed individuals not on ART. Here we quantified whether a pair represented a transmission pair independent of who infected whom for combination of parameters 1. NaN = no TP was observed.

| **Parameter / Tree / Perc.** | **Sensitivity** | **Specificity** | **Precision** |
| --- | --- | --- | --- |
| Group 1 / True trees / 5 | 0.022 / 0 | 1 / 1 | 0.8 / NaN |
| Group 1 / 1,000bp / 5 | 0.041 / 0.006 | 1 / 1 | 0.778 / 1 |
| Group 1 / 10,000bp / 5 | 0.028 / 0 | 1 / 1 | 0.833 / NaN |
| Group 1 / True trees / 10 | 0.016 / 0.006 | 0.999 / 1 | 0.706 / 0.714 |
| Group 1 / 1,000bp / 10 | 0.035 / 0.012 | 0.999 / 1 | 0.75 / 0.889 |
| Group 1 / 10,000bp / 10 | 0.017 / 0.007 | 1 / 1 | 0.765 / 0.714 |
| Group 1 / True trees / 20 | 0.019 / 0.005 | 0.999 / 1 | 0.742 / 0.857 |
| Group 1 / 1,000bp / 20 | 0.025 / 0.009 | 0.999 / 1 | 0.714 / 0.76 |
| Group 1 / 10,000bp / 20 | 0.019 / 0.004 | 0.999 / 1 | 0.703 / 0.833 |
| Group 1 / True trees / 30 | 0.021 / 0.004 | 0.998 / 1 | 0.73 / 0.808 |
| Group 1 / 1,000bp / 30 | 0.028 / 0.008 | 0.998 / 0.999 | 0.679 / 0.721 |
| Group 1 / 10,000bp / 30 | 0.021 / 0.005 | 0.998 / 1 | 0.729 / 0.778 |
| Group 1 / True trees / 40 | 0.019 / 0.006 | 0.998 / 1 | 0.697 / 0.8 |
| Group 1 / 1,000bp / 40 | 0.026 / 0.008 | 0.997 / 0.999 | 0.65 / 0.733 |
| Group 1 / 10,000bp / 40 | 0.021 / 0.006 | 0.997 / 1 | 0.704 / 0.77 |
| Group 1 / True trees / 50 | 0.021 / 0.005 | 0.997 / 1 | 0.722 / 0.821 |
| Group 1 / 1,000bp / 50 | 0.027 / 0.008 | 0.996 / 0.999 | 0.678 / 0.761 |
| Group 1 / 10,000bp / 50 | 0.021 / 0.006 | 0.997 / 0.999 | 0.729 / 0.783 |
| Group 1 / True trees / 60 | 0.024 / 0.006 | 0.997 / 0.999 | 0.782 / 0.81 |
| Group 1 / 1,000bp / 60 | 0.034 / 0.011 | 0.996 / 0.999 | 0.754 / 0.805 |
| Group 1 / 10,000bp / 60 | 0.026 / 0.006 | 0.997 / 0.999 | 0.782 / 0.842 |
| Group 1 / True trees / 70 | 0.024 / 0.006 | 0.997 / 0.999 | 0.793 / 0.86 |
| Group 1 / 1,000bp / 70 | 0.032 / 0.01 | 0.996 / 0.999 | 0.747 / 0.795 |
| Group 1 / 10,000bp / 70 | 0.025 / 0.007 | 0.997 / 0.999 | 0.794 / 0.847 |
| Group 1 / True trees / 80 | 0.026 / 0.007 | 0.996 / 0.999 | 0.799 / 0.849 |
| Group 1 / 1,000bp / 80 | 0.035 / 0.012 | 0.995 / 0.999 | 0.758 / 0.802 |
| Group 1 / 10,000bp / 80 | 0.027 / 0.007 | 0.996 / 0.999 | 0.795 / 0.85 |
| Group 1 / True trees / 90 | 0.026 / 0.007 | 0.996 / 0.999 | 0.825 / 0.891 |
| Group 1 / 1,000bp / 90 | 0.036 / 0.011 | 0.994 / 0.999 | 0.77 / 0.801 |
| Group 1 / 10,000bp / 90 | 0.028 / 0.007 | 0.996 / 0.999 | 0.818 / 0.869 |

**Table S11: Infector probability results for sampling strategy 1 for an average of 500 migrants per year to and from *region* for parameter group 2.** Sensitivity, specificity and precision for infector probabilities for two thresholds (80% and 90%) for true trees and ML trees estimated with 1,000bp and 10,000bp for different sampling depth of 5 to 90% (perc.) of diagnosed individuals not on ART. Here we quantified whether a pair represented a transmission pair independent of who infected whom for combination of parameters 2.

| **Parameter / Tree / Perc.** | **Sensitivity** | **Specificity** | **Precision** |
| --- | --- | --- | --- |
| Group 2 / True trees / 5 | 0.005 / 0.005 | 1 / 1 | 0.667 / 1 |
| Group 2 / 1,000bp / 5 | 0.006 / 0.006 | 1 / 1 | 0.667 / 1 |
| Group 2 / 10,000bp / 5 | 0.008 / 0.005 | 1 / 1 | 0.75 / 1 |
| Group 2 / True trees / 10 | 0.01 / 0.002 | 1 / 1 | 0.682 / 0.5 |
| Group 2 / 1,000bp / 10 | 0.015 / 0.004 | 0.999 / 1 | 0.576 / 0.556 |
| Group 2 / 10,000bp / 10 | 0.01 / 0.002 | 1 / 1 | 0.714 / 0.6 |
| Group 2 / True trees / 20 | 0.01 / 0.001 | 0.999 / 1 | 0.764 / 0.7 |
| Group 2 / 1,000bp / 20 | 0.017 / 0.003 | 0.999 / 1 | 0.738 / 0.619 |
| Group 2 / 10,000bp / 20 | 0.011 / 0.001 | 0.999 / 1 | 0.753 / 0.7 |
| Group 2 / True trees / 30 | 0.012 / 0.003 | 0.999 / 1 | 0.78 / 0.925 |
| Group 2 / 1,000bp / 30 | 0.018 / 0.005 | 0.999 / 1 | 0.773 / 0.849 |
| Group 2 / 10,000bp / 30 | 0.013 / 0.004 | 0.999 / 1 | 0.777 / 0.844 |
| Group 2 / True trees / 40 | 0.012 / 0.003 | 0.999 / 1 | 0.769 / 0.806 |
| Group 2 / 1,000bp / 40 | 0.019 / 0.006 | 0.998 / 1 | 0.713 / 0.786 |
| Group 2 / 10,000bp / 40 | 0.013 / 0.003 | 0.998 / 1 | 0.733 / 0.811 |
| Group 2 / True trees / 50 | 0.015 / 0.003 | 0.998 / 1 | 0.825 / 0.864 |
| Group 2 / 1,000bp / 50 | 0.021 / 0.006 | 0.998 / 1 | 0.79 / 0.848 |
| Group 2 / 10,000bp / 50 | 0.015 / 0.004 | 0.998 / 1 | 0.823 / 0.86 |
| Group 2 / True trees / 60 | 0.014 / 0.004 | 0.998 / 0.999 | 0.784 / 0.814 |
| Group 2 / 1,000bp / 60 | 0.022 / 0.007 | 0.997 / 0.999 | 0.781 / 0.837 |
| Group 2 / 10,000bp / 60 | 0.016 / 0.004 | 0.998 / 1 | 0.808 / 0.841 |
| Group 2 / True trees / 70 | 0.016 / 0.005 | 0.998 / 1 | 0.86 / 0.915 |
| Group 2 / 1,000bp / 70 | 0.022 / 0.006 | 0.997 / 0.999 | 0.79 / 0.846 |
| Group 2 / 10,000bp / 70 | 0.017 / 0.005 | 0.998 / 0.999 | 0.828 / 0.861 |
| Group 2 / True trees / 80 | 0.016 / 0.004 | 0.997 / 0.999 | 0.831 / 0.851 |
| Group 2 / 1,000bp / 80 | 0.024 / 0.007 | 0.996 / 0.999 | 0.79 / 0.871 |
| Group 2 / 10,000bp / 80 | 0.018 / 0.004 | 0.997 / 0.999 | 0.83 / 0.861 |
| Group 2 / True trees / 90 | 0.018 / 0.005 | 0.997 / 1 | 0.857 / 0.901 |
| Group 2 / 1,000bp / 90 | 0.026 / 0.009 | 0.997 / 0.999 | 0.827 / 0.86 |
| Group 2 / 10,000bp / 90 | 0.019 / 0.005 | 0.997 / 0.999 | 0.85 / 0.886 |

**Table S12: Infector probability results for sampling strategy 1 for an average of 750 migrants per year to and from *region* for parameter group 1.** Sensitivity, specificity and precision for infector probabilities for two thresholds (80% and 90%) for true trees and ML trees estimated with 1,000bp and 10,000bp for different sampling depth of 5 to 90% (perc.) of diagnosed individuals not on ART. Here we quantified whether a pair represented a transmission pair independent of who infected whom for combination of parameters 1.

| **Parameter / Tree / Perc.** | **Sensitivity** | **Specificity** | **Precision** |
| --- | --- | --- | --- |
| Group 1 / True trees / 5 | 0.026 / 0.009 | 1 / 1 | 0.75 / 1 |
| Group 1 / 1,000bp / 5 | 0.028 / 0.009 | 1 / 1 | 0.75 / 1 |
| Group 1 / 10,000bp / 5 | 0.029 / 0.01 | 1 / 1 | 1 / 1 |
| Group 1 / True trees / 10 | 0.009 / 0.003 | 1 / 1 | 0.714 / 1 |
| Group 1 / 1,000bp / 10 | 0.017 / 0.004 | 0.999 / 1 | 0.6 / 0.667 |
| Group 1 / 10,000bp / 10 | 0.011 / 0.002 | 1 / 1 | 0.75 / 0.5 |
| Group 1 / True trees / 20 | 0.02 / 0.005 | 0.999 / 1 | 0.78 / 0.706 |
| Group 1 / 1,000bp / 20 | 0.022 / 0.009 | 0.998 / 1 | 0.647 / 0.75 |
| Group 1 / 10,000bp / 20 | 0.02 / 0.005 | 0.999 / 1 | 0.763 / 0.688 |
| Group 1 / True trees / 30 | 0.016 / 0.004 | 0.999 / 1 | 0.742 / 0.864 |
| Group 1 / 1,000bp / 30 | 0.021 / 0.009 | 0.998 / 1 | 0.648 / 0.85 |
| Group 1 / 10,000bp / 30 | 0.019 / 0.004 | 0.999 / 1 | 0.718 / 0.864 |
| Group 1 / True trees / 40 | 0.019 / 0.005 | 0.998 / 1 | 0.749 / 0.72 |
| Group 1 / 1,000bp / 40 | 0.028 / 0.009 | 0.997 / 0.999 | 0.699 / 0.697 |
| Group 1 / 10,000bp / 40 | 0.02 / 0.006 | 0.998 / 1 | 0.718 / 0.75 |
| Group 1 / True trees / 50 | 0.023 / 0.006 | 0.998 / 1 | 0.759 / 0.831 |
| Group 1 / 1,000bp / 50 | 0.033 / 0.011 | 0.997 / 0.999 | 0.718 / 0.84 |
| Group 1 / 10,000bp / 50 | 0.024 / 0.007 | 0.998 / 1 | 0.757 / 0.821 |
| Group 1 / True trees / 60 | 0.022 / 0.005 | 0.998 / 1 | 0.807 / 0.885 |
| Group 1 / 1,000bp / 60 | 0.032 / 0.01 | 0.996 / 0.999 | 0.735 / 0.81 |
| Group 1 / 10,000bp / 60 | 0.024 / 0.006 | 0.998 / 1 | 0.783 / 0.867 |
| Group 1 / True trees / 70 | 0.022 / 0.006 | 0.997 / 1 | 0.775 / 0.864 |
| Group 1 / 1,000bp / 70 | 0.03 / 0.009 | 0.996 / 0.999 | 0.721 / 0.81 |
| Group 1 / 10,000bp / 70 | 0.024 / 0.006 | 0.997 / 1 | 0.779 / 0.875 |
| Group 1 / True trees / 80 | 0.024 / 0.006 | 0.997 / 0.999 | 0.796 / 0.83 |
| Group 1 / 1,000bp / 80 | 0.034 / 0.011 | 0.995 / 0.999 | 0.757 / 0.806 |
| Group 1 / 10,000bp / 80 | 0.025 / 0.007 | 0.997 / 0.999 | 0.785 / 0.844 |
| Group 1 / True trees / 90 | 0.027 / 0.007 | 0.997 / 1 | 0.837 / 0.901 |
| Group 1 / 1,000bp / 90 | 0.037 / 0.012 | 0.996 / 0.999 | 0.801 / 0.834 |
| Group 1 / 10,000bp / 90 | 0.027 / 0.008 | 0.997 / 1 | 0.83 / 0.902 |

**Table S13: Infector probability results for sampling strategy 1 for an average of 750 migrants per year to and from *region* for parameter group 2.** Sensitivity, specificity and precision for infector probabilities for two thresholds (80% and 90%) for true trees and ML trees estimated with 1,000bp and 10,000bp for different sampling depth of 5 to 90% (perc.) of diagnosed individuals not on ART. Here we quantified whether a pair represented a transmission pair independent of who infected whom for combination of parameters 2.

| **Parameter / Tree / Perc.** | **Sensitivity** | **Specificity** | **Precision** |
| --- | --- | --- | --- |
| Group 2 / True trees / 5 | 0.008 / 0.005 | 1 / 1 | 0.6 / 1 |
| Group 2 / 1,000bp / 5 | 0.01 / 0.006 | 1 / 1 | 0.5 / 1 |
| Group 2 / 10,000bp / 5 | 0.008 / 0.005 | 1 / 1 | 0.75 / 1 |
| Group 2 / True trees / 10 | 0.008 / 0.003 | 1 / 1 | 0.769 / 0.667 |
| Group 2 / 1,000bp / 10 | 0.016 / 0.003 | 1 / 1 | 0.696 / 0.5 |
| Group 2 / 10,000bp / 10 | 0.009 / 0.003 | 1 / 1 | 0.786 / 0.667 |
| Group 2 / True trees / 20 | 0.011 / 0.003 | 0.999 / 1 | 0.754 / 0.875 |
| Group 2 / 1,000bp / 20 | 0.015 / 0.005 | 0.999 / 1 | 0.713 / 0.792 |
| Group 2 / 10,000bp / 20 | 0.012 / 0.003 | 0.999 / 1 | 0.761 / 0.812 |
| Group 2 / True trees / 30 | 0.011 / 0.003 | 0.999 / 1 | 0.696 / 0.8 |
| Group 2 / 1,000bp / 30 | 0.016 / 0.005 | 0.998 / 1 | 0.665 / 0.764 |
| Group 2 / 10,000bp / 30 | 0.011 / 0.004 | 0.999 / 1 | 0.71 / 0.767 |
| Group 2 / True trees / 40 | 0.013 / 0.003 | 0.999 / 1 | 0.765 / 0.807 |
| Group 2 / 1,000bp / 40 | 0.019 / 0.006 | 0.998 / 1 | 0.734 / 0.804 |
| Group 2 / 10,000bp / 40 | 0.014 / 0.004 | 0.998 / 1 | 0.755 / 0.809 |
| Group 2 / True trees / 50 | 0.013 / 0.003 | 0.998 / 1 | 0.758 / 0.84 |
| Group 2 / 1,000bp / 50 | 0.021 / 0.007 | 0.998 / 0.999 | 0.76 / 0.817 |
| Group 2 / 10,000bp / 50 | 0.014 / 0.004 | 0.998 / 1 | 0.751 / 0.824 |
| Group 2 / True trees / 60 | 0.013 / 0.003 | 0.998 / 1 | 0.804 / 0.822 |
| Group 2 / 1,000bp / 60 | 0.019 / 0.006 | 0.998 / 0.999 | 0.749 / 0.787 |
| Group 2 / 10,000bp / 60 | 0.014 / 0.004 | 0.998 / 1 | 0.787 / 0.843 |
| Group 2 / True trees / 70 | 0.014 / 0.004 | 0.998 / 1 | 0.816 / 0.885 |
| Group 2 / 1,000bp / 70 | 0.021 / 0.006 | 0.997 / 0.999 | 0.781 / 0.842 |
| Group 2 / 10,000bp / 70 | 0.015 / 0.004 | 0.998 / 1 | 0.813 / 0.865 |
| Group 2 / True trees / 80 | 0.016 / 0.004 | 0.997 / 0.999 | 0.821 / 0.823 |
| Group 2 / 1,000bp / 80 | 0.024 / 0.007 | 0.997 / 0.999 | 0.777 / 0.84 |
| Group 2 / 10,000bp / 80 | 0.017 / 0.004 | 0.998 / 0.999 | 0.82 / 0.833 |
| Group 2 / True trees / 90 | 0.017 / 0.004 | 0.998 / 1 | 0.847 / 0.905 |
| Group 2 / 1,000bp / 90 | 0.026 / 0.008 | 0.997 / 0.999 | 0.821 / 0.882 |
| Group 2 / 10,000bp / 90 | 0.019 / 0.005 | 0.997 / 1 | 0.847 / 0.88 |

**Table S14: Infector probability results for sampling strategy 2 for an average of 250 migrants per year to and from *region* for parameter group 1.** Sensitivity, specificity and precision for infector probabilities for two thresholds (80% and 90%) for true trees and ML trees estimated with 1,000bp and 10,000bp for different sampling depth of 5 to 90% (perc.) of diagnosed individuals independent of ART-status. Here we quantified whether a pair represented a transmission pair independent of who infected whom for combination of parameters 1.

| **Parameter / Tree / Perc.** | **Sensitivity** | **Specificity** | **Precision** |
| --- | --- | --- | --- |
| Group 1 / True trees / 5 | 0.007 / 0.004 | 1 / 1 | 0.778 / 1 |
| Group 1 / 1,000bp / 5 | 0.012 / 0.003 | 1 / 1 | 0.786 / 1 |
| Group 1 / 10,000bp / 5 | 0.006 / 0.003 | 1 / 1 | 0.75 / 1 |
| Group 1 / True trees / 10 | 0.005 / 0.001 | 0.999 / 1 | 0.656 / 0.625 |
| Group 1 / 1,000bp / 10 | 0.007 / 0.002 | 0.999 / 1 | 0.561 / 0.462 |
| Group 1 / 10,000bp / 10 | 0.006 / 0.001 | 0.999 / 1 | 0.647 / 0.625 |
| Group 1 / True trees / 20 | 0.005 / 0.001 | 0.999 / 1 | 0.724 / 0.714 |
| Group 1 / 1,000bp / 20 | 0.007 / 0.002 | 0.999 / 1 | 0.697 / 0.621 |
| Group 1 / 10,000bp / 20 | 0.006 / 0.001 | 0.999 / 1 | 0.725 / 0.75 |
| Group 1 / True trees / 30 | 0.007 / 0.001 | 0.999 / 1 | 0.745 / 0.927 |
| Group 1 / 1,000bp / 30 | 0.01 / 0.003 | 0.998 / 1 | 0.707 / 0.844 |
| Group 1 / 10,000bp / 30 | 0.007 / 0.002 | 0.998 / 1 | 0.74 / 0.87 |
| Group 1 / True trees / 40 | 0.008 / 0.002 | 0.998 / 1 | 0.803 / 0.859 |
| Group 1 / 1,000bp / 40 | 0.011 / 0.003 | 0.998 / 1 | 0.758 / 0.811 |
| Group 1 / 10,000bp / 40 | 0.009 / 0.002 | 0.998 / 1 | 0.801 / 0.813 |
| Group 1 / True trees / 50 | 0.009 / 0.002 | 0.998 / 0.999 | 0.835 / 0.803 |
| Group 1 / 1,000bp / 50 | 0.013 / 0.003 | 0.998 / 0.999 | 0.81 / 0.826 |
| Group 1 / 10,000bp / 50 | 0.01 / 0.002 | 0.998 / 1 | 0.83 / 0.824 |
| Group 1 / True trees / 60 | 0.009 / 0.002 | 0.997 / 0.999 | 0.819 / 0.854 |
| Group 1 / 1,000bp / 60 | 0.013 / 0.004 | 0.997 / 0.999 | 0.811 / 0.852 |
| Group 1 / 10,000bp / 60 | 0.01 / 0.003 | 0.997 / 0.999 | 0.826 / 0.861 |
| Group 1 / True trees / 70 | 0.01 / 0.002 | 0.997 / 0.999 | 0.851 / 0.864 |
| Group 1 / 1,000bp / 70 | 0.014 / 0.004 | 0.996 / 0.999 | 0.821 / 0.838 |
| Group 1 / 10,000bp / 70 | 0.01 / 0.003 | 0.997 / 0.999 | 0.842 / 0.855 |
| Group 1 / True trees / 80 | 0.011 / 0.002 | 0.996 / 0.999 | 0.871 / 0.871 |
| Group 1 / 1,000bp / 80 | 0.015 / 0.004 | 0.996 / 0.999 | 0.839 / 0.868 |
| Group 1 / 10,000bp / 80 | 0.011 / 0.003 | 0.996 / 0.999 | 0.862 / 0.902 |
| Group 1 / True trees / 90 | 0.011 / 0.003 | 0.996 / 0.999 | 0.881 / 0.892 |
| Group 1 / 1,000bp / 90 | 0.016 / 0.005 | 0.996 / 0.999 | 0.853 / 0.867 |
| Group 1 / 10,000bp / 90 | 0.012 / 0.003 | 0.996 / 0.999 | 0.88 / 0.896 |

**Table S15: Infector probability results for sampling strategy 2 for an average of 250 migrants per year to and from *region* for parameter group 2.** Sensitivity, specificity and precision for infector probabilities for two thresholds (80% and 90%) for true trees and ML trees estimated with 1,000bp and 10,000bp for different sampling depth of 5 to 90% (perc.) of diagnosed individuals independent of ART-status. Here we quantified whether a pair represented a transmission pair independent of who infected whom for combination of parameters 2.

| **Parameter / Tree / Perc.** | **Sensitivity** | **Specificity** | **Precision** |
| --- | --- | --- | --- |
| Group 2 / True trees / 5 | 0.008 / 0.003 | 1 / 1 | 0.923 / 1 |
| Group 2 / 1,000bp / 5 | 0.009 / 0.002 | 1 / 1 | 0.632 / 1 |
| Group 2 / 10,000bp / 5 | 0.008 / 0.003 | 1 / 1 | 0.8 / 1 |
| Group 2 / True trees / 10 | 0.004 / 0.001 | 1 / 1 | 0.618 / 0.667 |
| Group 2 / 1,000bp / 10 | 0.006 / 0.001 | 0.999 / 1 | 0.617 / 0.545 |
| Group 2 / 10,000bp / 10 | 0.005 / 0.001 | 1 / 1 | 0.744 / 0.714 |
| Group 2 / True trees / 20 | 0.006 / 0.002 | 0.999 / 1 | 0.704 / 0.854 |
| Group 2 / 1,000bp / 20 | 0.01 / 0.003 | 0.999 / 1 | 0.736 / 0.733 |
| Group 2 / 10,000bp / 20 | 0.007 / 0.002 | 0.999 / 1 | 0.71 / 0.848 |
| Group 2 / True trees / 30 | 0.006 / 0.001 | 0.999 / 1 | 0.726 / 0.727 |
| Group 2 / 1,000bp / 30 | 0.009 / 0.002 | 0.998 / 1 | 0.719 / 0.702 |
| Group 2 / 10,000bp / 30 | 0.006 / 0.002 | 0.998 / 1 | 0.712 / 0.75 |
| Group 2 / True trees / 40 | 0.007 / 0.002 | 0.998 / 1 | 0.811 / 0.857 |
| Group 2 / 1,000bp / 40 | 0.011 / 0.003 | 0.998 / 1 | 0.783 / 0.848 |
| Group 2 / 10,000bp / 40 | 0.008 / 0.002 | 0.999 / 1 | 0.824 / 0.847 |
| Group 2 / True trees / 50 | 0.009 / 0.002 | 0.998 / 1 | 0.855 / 0.892 |
| Group 2 / 1,000bp / 50 | 0.012 / 0.004 | 0.998 / 0.999 | 0.815 / 0.834 |
| Group 2 / 10,000bp / 50 | 0.009 / 0.002 | 0.998 / 1 | 0.847 / 0.852 |
| Group 2 / True trees / 60 | 0.008 / 0.002 | 0.998 / 0.999 | 0.842 / 0.845 |
| Group 2 / 1,000bp / 60 | 0.012 / 0.003 | 0.997 / 0.999 | 0.819 / 0.816 |
| Group 2 / 10,000bp / 60 | 0.009 / 0.002 | 0.998 / 1 | 0.835 / 0.869 |
| Group 2 / True trees / 70 | 0.009 / 0.002 | 0.997 / 0.999 | 0.872 / 0.875 |
| Group 2 / 1,000bp / 70 | 0.013 / 0.004 | 0.997 / 0.999 | 0.84 / 0.877 |
| Group 2 / 10,000bp / 70 | 0.009 / 0.002 | 0.997 / 0.999 | 0.859 / 0.876 |
| Group 2 / True trees / 80 | 0.01 / 0.003 | 0.997 / 0.999 | 0.88 / 0.892 |
| Group 2 / 1,000bp / 80 | 0.014 / 0.004 | 0.997 / 0.999 | 0.857 / 0.892 |
| Group 2 / 10,000bp / 80 | 0.01 / 0.003 | 0.997 / 0.999 | 0.889 / 0.918 |
| Group 2 / True trees / 90 | 0.011 / 0.002 | 0.996 / 0.999 | 0.903 / 0.928 |
| Group 2 / 1,000bp / 90 | 0.016 / 0.004 | 0.996 / 0.999 | 0.867 / 0.86 |
| Group 2 / 10,000bp / 90 | 0.011 / 0.003 | 0.997 / 0.999 | 0.903 / 0.925 |

**Table S16: Infector probability results for sampling strategy 2 for an average of 500 migrants per year to and from *region* for parameter group 1.** Sensitivity, specificity and precision for infector probabilities for two thresholds (80% and 90%) for true trees and ML trees estimated with 1,000bp and 10,000bp for different sampling depth of 5 to 90% (perc.) of diagnosed individuals independent of ART-status. Here we quantified whether a pair represented a transmission pair independent of who infected whom for combination of parameters 1.

| **Parameter / Tree / Perc.** | **Sensitivity** | **Specificity** | **Precision** |
| --- | --- | --- | --- |
| Group 1 / True trees / 5 | 0.008 / 0.003 | 1 / 1 | 0.778 / 1 |
| Group 1 / 1,000bp / 5 | 0.01 / 0.006 | 1 / 1 | 0.8 / 1 |
| Group 1 / 10,000bp / 5 | 0.008 / 0.003 | 1 / 1 | 0.7 / 0.75 |
| Group 1 / True trees / 10 | 0.005 / 0.001 | 0.999 / 1 | 0.615 / 1 |
| Group 1 / 1,000bp / 10 | 0.008 / 0.002 | 0.999 / 1 | 0.639 / 0.625 |
| Group 1 / 10,000bp / 10 | 0.006 / 0.001 | 0.999 / 1 | 0.625 / 0.667 |
| Group 1 / True trees / 20 | 0.006 / 0.001 | 0.999 / 1 | 0.79 / 0.929 |
| Group 1 / 1,000bp / 20 | 0.009 / 0.002 | 0.999 / 1 | 0.706 / 0.792 |
| Group 1 / 10,000bp / 20 | 0.007 / 0.001 | 0.999 / 1 | 0.802 / 0.875 |
| Group 1 / True trees / 30 | 0.007 / 0.002 | 0.999 / 1 | 0.776 / 0.755 |
| Group 1 / 1,000bp / 30 | 0.012 / 0.003 | 0.998 / 1 | 0.761 / 0.81 |
| Group 1 / 10,000bp / 30 | 0.007 / 0.002 | 0.999 / 1 | 0.754 / 0.765 |
| Group 1 / True trees / 40 | 0.008 / 0.002 | 0.999 / 1 | 0.796 / 0.873 |
| Group 1 / 1,000bp / 40 | 0.012 / 0.003 | 0.998 / 1 | 0.74 / 0.808 |
| Group 1 / 10,000bp / 40 | 0.008 / 0.002 | 0.998 / 1 | 0.782 / 0.863 |
| Group 1 / True trees / 50 | 0.009 / 0.002 | 0.998 / 1 | 0.825 / 0.857 |
| Group 1 / 1,000bp / 50 | 0.013 / 0.004 | 0.998 / 1 | 0.795 / 0.859 |
| Group 1 / 10,000bp / 50 | 0.01 / 0.002 | 0.999 / 1 | 0.848 / 0.842 |
| Group 1 / True trees / 60 | 0.008 / 0.002 | 0.998 / 1 | 0.83 / 0.839 |
| Group 1 / 1,000bp / 60 | 0.013 / 0.003 | 0.997 / 0.999 | 0.791 / 0.845 |
| Group 1 / 10,000bp / 60 | 0.01 / 0.002 | 0.998 / 1 | 0.842 / 0.895 |
| Group 1 / True trees / 70 | 0.01 / 0.002 | 0.997 / 1 | 0.843 / 0.884 |
| Group 1 / 1,000bp / 70 | 0.014 / 0.004 | 0.997 / 0.999 | 0.815 / 0.855 |
| Group 1 / 10,000bp / 70 | 0.011 / 0.003 | 0.998 / 1 | 0.848 / 0.909 |
| Group 1 / True trees / 80 | 0.01 / 0.002 | 0.997 / 0.999 | 0.86 / 0.859 |
| Group 1 / 1,000bp / 80 | 0.015 / 0.004 | 0.996 / 0.999 | 0.829 / 0.85 |
| Group 1 / 10,000bp / 80 | 0.011 / 0.003 | 0.997 / 0.999 | 0.861 / 0.869 |
| Group 1 / True trees / 90 | 0.011 / 0.003 | 0.997 / 1 | 0.872 / 0.924 |
| Group 1 / 1,000bp / 90 | 0.016 / 0.005 | 0.996 / 0.999 | 0.839 / 0.868 |
| Group 1 / 10,000bp / 90 | 0.012 / 0.003 | 0.997 / 0.999 | 0.866 / 0.894 |

**Table S17: Infector probability results for sampling strategy 2 for an average of 500 migrants per year to and from *region* for parameter group 2.** Sensitivity, specificity and precision for infector probabilities for two thresholds (80% and 90%) for true trees and ML trees estimated with 1,000bp and 10,000bp for different sampling depth of 5 to 90% (perc.) of diagnosed individuals independent of ART-status. Here we quantified whether a pair represented a transmission pair independent of who infected whom for combination of parameters 2. NaN = no TP was observed.

| **Parameter / Tree / Perc.** | **Sensitivity** | **Specificity** | **Precision** |
| --- | --- | --- | --- |
| Group 2 / True trees / 5 | 0.004 / 0 | 1 / 1 | 0.833 / NaN |
| Group 2 / 1,000bp / 5 | 0.007 / 0 | 1 / 1 | 0.667 / 0 |
| Group 2 / 10,000bp / 5 | 0.005 / 0 | 1 / 1 | 0.857 / NaN |
| Group 2 / True trees / 10 | 0.003 / 0.001 | 1 / 1 | 0.737 / 0.75 |
| Group 2 / 1,000bp / 10 | 0.006 / 0.001 | 0.999 / 1 | 0.629 / 0.429 |
| Group 2 / 10,000bp / 10 | 0.003 / 0 | 1 / 1 | 0.684 / 0.667 |
| Group 2 / True trees / 20 | 0.006 / 0.002 | 1 / 1 | 0.83 / 0.885 |
| Group 2 / 1,000bp / 20 | 0.01 / 0.003 | 0.999 / 1 | 0.83 / 0.822 |
| Group 2 / 10,000bp / 20 | 0.006 / 0.002 | 0.999 / 1 | 0.783 / 0.893 |
| Group 2 / True trees / 30 | 0.006 / 0.002 | 0.999 / 1 | 0.785 / 0.831 |
| Group 2 / 1,000bp / 30 | 0.01 / 0.003 | 0.999 / 1 | 0.751 / 0.8 |
| Group 2 / 10,000bp / 30 | 0.007 / 0.002 | 0.999 / 1 | 0.777 / 0.839 |
| Group 2 / True trees / 40 | 0.007 / 0.002 | 0.999 / 1 | 0.781 / 0.855 |
| Group 2 / 1,000bp / 40 | 0.011 / 0.003 | 0.998 / 1 | 0.767 / 0.775 |
| Group 2 / 10,000bp / 40 | 0.008 / 0.002 | 0.999 / 1 | 0.819 / 0.866 |
| Group 2 / True trees / 50 | 0.008 / 0.002 | 0.998 / 1 | 0.811 / 0.84 |
| Group 2 / 1,000bp / 50 | 0.01 / 0.003 | 0.998 / 1 | 0.768 / 0.785 |
| Group 2 / 10,000bp / 50 | 0.008 / 0.002 | 0.998 / 1 | 0.81 / 0.875 |
| Group 2 / True trees / 60 | 0.008 / 0.002 | 0.998 / 1 | 0.833 / 0.858 |
| Group 2 / 1,000bp / 60 | 0.012 / 0.003 | 0.998 / 0.999 | 0.833 / 0.824 |
| Group 2 / 10,000bp / 60 | 0.009 / 0.002 | 0.998 / 1 | 0.836 / 0.837 |
| Group 2 / True trees / 70 | 0.009 / 0.002 | 0.998 / 1 | 0.882 / 0.922 |
| Group 2 / 1,000bp / 70 | 0.013 / 0.004 | 0.998 / 0.999 | 0.85 / 0.867 |
| Group 2 / 10,000bp / 70 | 0.009 / 0.002 | 0.998 / 1 | 0.871 / 0.907 |
| Group 2 / True trees / 80 | 0.008 / 0.002 | 0.998 / 1 | 0.873 / 0.902 |
| Group 2 / 1,000bp / 80 | 0.013 / 0.004 | 0.998 / 1 | 0.852 / 0.892 |
| Group 2 / 10,000bp / 80 | 0.009 / 0.002 | 0.998 / 1 | 0.875 / 0.896 |
| Group 2 / True trees / 90 | 0.01 / 0.002 | 0.998 / 1 | 0.905 / 0.947 |
| Group 2 / 1,000bp / 90 | 0.014 / 0.004 | 0.997 / 0.999 | 0.871 / 0.876 |
| Group 2 / 10,000bp / 90 | 0.01 / 0.002 | 0.998 / 1 | 0.888 / 0.929 |

**Table S18: Infector probability results for sampling strategy 2 for an average of 750 migrants per year to and from *region* for parameter group 1.** Sensitivity, specificity and precision for infector probabilities for two thresholds (80% and 90%) for true trees and ML trees estimated with 1,000bp and 10,000bp for different sampling depth of 5 to 90% (perc.) of diagnosed individuals independent of ART-status. Here we quantified whether a pair represented a transmission pair independent of who infected whom for combination of parameters 1.

| **Parameter / Tree / Perc.** | **Sensitivity** | **Specificity** | **Precision** |
| --- | --- | --- | --- |
| Group 1 / True trees / 5 | 0.007 / 0.001 | 1 / 1 | 0.714 / 1 |
| Group 1 / 1,000bp / 5 | 0.006 / 0 | 1 / 1 | 0.571 / 0 |
| Group 1 / 10,000bp / 5 | 0.007 / 0.001 | 1 / 1 | 0.714 / 1 |
| Group 1 / True trees / 10 | 0.004 / 0.002 | 1 / 1 | 0.556 / 1 |
| Group 1 / 1,000bp / 10 | 0.006 / 0.003 | 0.999 / 1 | 0.5 / 0.889 |
| Group 1 / 10,000bp / 10 | 0.005 / 0.002 | 0.999 / 1 | 0.571 / 1 |
| Group 1 / True trees / 20 | 0.008 / 0.003 | 0.999 / 1 | 0.802 / 0.885 |
| Group 1 / 1,000bp / 20 | 0.012 / 0.004 | 0.999 / 1 | 0.758 / 0.889 |
| Group 1 / 10,000bp / 20 | 0.009 / 0.003 | 0.999 / 1 | 0.777 / 0.875 |
| Group 1 / True trees / 30 | 0.007 / 0.001 | 0.999 / 1 | 0.839 / 0.758 |
| Group 1 / 1,000bp / 30 | 0.011 / 0.002 | 0.999 / 1 | 0.763 / 0.735 |
| Group 1 / 10,000bp / 30 | 0.008 / 0.002 | 0.999 / 1 | 0.812 / 0.757 |
| Group 1 / True trees / 40 | 0.009 / 0.002 | 0.999 / 1 | 0.824 / 0.813 |
| Group 1 / 1,000bp / 40 | 0.012 / 0.003 | 0.998 / 1 | 0.768 / 0.767 |
| Group 1 / 10,000bp / 40 | 0.009 / 0.002 | 0.999 / 1 | 0.804 / 0.818 |
| Group 1 / True trees / 50 | 0.008 / 0.002 | 0.999 / 1 | 0.809 / 0.859 |
| Group 1 / 1,000bp / 50 | 0.012 / 0.003 | 0.998 / 0.999 | 0.773 / 0.744 |
| Group 1 / 10,000bp / 50 | 0.009 / 0.002 | 0.999 / 1 | 0.819 / 0.835 |
| Group 1 / True trees / 60 | 0.009 / 0.002 | 0.998 / 0.999 | 0.836 / 0.805 |
| Group 1 / 1,000bp / 60 | 0.013 / 0.004 | 0.997 / 0.999 | 0.774 / 0.818 |
| Group 1 / 10,000bp / 60 | 0.01 / 0.002 | 0.998 / 0.999 | 0.794 / 0.768 |
| Group 1 / True trees / 70 | 0.01 / 0.002 | 0.998 / 1 | 0.827 / 0.887 |
| Group 1 / 1,000bp / 70 | 0.014 / 0.004 | 0.997 / 0.999 | 0.802 / 0.842 |
| Group 1 / 10,000bp / 70 | 0.01 / 0.003 | 0.998 / 1 | 0.82 / 0.894 |
| Group 1 / True trees / 80 | 0.01 / 0.002 | 0.997 / 0.999 | 0.84 / 0.865 |
| Group 1 / 1,000bp / 80 | 0.014 / 0.004 | 0.997 / 0.999 | 0.823 / 0.858 |
| Group 1 / 10,000bp / 80 | 0.011 / 0.003 | 0.998 / 1 | 0.85 / 0.922 |
| Group 1 / True trees / 90 | 0.012 / 0.003 | 0.997 / 0.999 | 0.866 / 0.88 |
| Group 1 / 1,000bp / 90 | 0.017 / 0.005 | 0.996 / 0.999 | 0.829 / 0.867 |
| Group 1 / 10,000bp / 90 | 0.012 / 0.003 | 0.997 / 0.999 | 0.868 / 0.879 |

**Table S19: Infector probability results for sampling strategy 2 for an average of 750 migrants per year to and from *region* for parameter group 2.** Sensitivity, specificity and precision for infector probabilities for two thresholds (80% and 90%) for true trees and ML trees estimated with 1,000bp and 10,000bp for different sampling depth of 5 to 90% (perc.) of diagnosed individuals independent of ART-status. Here we quantified whether a pair represented a transmission pair independent of who infected whom for combination of parameters 2. NaN = no TP was observed.

| **Parameter / Tree / Perc.** | **Sensitivity** | **Specificity** | **Precision** |
| --- | --- | --- | --- |
| Group 2 / True trees / 5 | 0.007 / 0 | 1 / 1 | 0.75 / NaN |
| Group 2 / 1,000bp / 5 | 0.01 / 0 | 1 / 1 | 0.727 / NaN |
| Group 2 / 10,000bp / 5 | 0.003 / 0 | 1 / 1 | 0.6 / NaN |
| Group 2 / True trees / 10 | 0.007 / 0.001 | 1 / 1 | 0.828 / 0.833 |
| Group 2 / 1,000bp / 10 | 0.011 / 0.003 | 1 / 1 | 0.8 / 0.909 |
| Group 2 / 10,000bp / 10 | 0.007 / 0.002 | 1 / 1 | 0.852 / 0.875 |
| Group 2 / True trees / 20 | 0.006 / 0.001 | 0.999 / 1 | 0.727 / 0.652 |
| Group 2 / 1,000bp / 20 | 0.009 / 0.002 | 0.999 / 1 | 0.713 / 0.786 |
| Group 2 / 10,000bp / 20 | 0.006 / 0.001 | 0.999 / 1 | 0.753 / 0.652 |
| Group 2 / True trees / 30 | 0.006 / 0.001 | 0.999 / 1 | 0.762 / 0.842 |
| Group 2 / 1,000bp / 30 | 0.01 / 0.003 | 0.999 / 1 | 0.722 / 0.736 |
| Group 2 / 10,000bp / 30 | 0.007 / 0.002 | 0.999 / 1 | 0.776 / 0.787 |
| Group 2 / True trees / 40 | 0.007 / 0.001 | 0.999 / 1 | 0.786 / 0.809 |
| Group 2 / 1,000bp / 40 | 0.011 / 0.003 | 0.999 / 1 | 0.764 / 0.793 |
| Group 2 / 10,000bp / 40 | 0.007 / 0.001 | 0.999 / 1 | 0.799 / 0.828 |
| Group 2 / True trees / 50 | 0.007 / 0.002 | 0.999 / 1 | 0.797 / 0.822 |
| Group 2 / 1,000bp / 50 | 0.011 / 0.003 | 0.998 / 1 | 0.765 / 0.768 |
| Group 2 / 10,000bp / 50 | 0.008 / 0.002 | 0.999 / 1 | 0.829 / 0.891 |
| Group 2 / True trees / 60 | 0.008 / 0.002 | 0.999 / 1 | 0.84 / 0.846 |
| Group 2 / 1,000bp / 60 | 0.012 / 0.003 | 0.998 / 1 | 0.778 / 0.812 |
| Group 2 / 10,000bp / 60 | 0.009 / 0.002 | 0.998 / 1 | 0.814 / 0.829 |
| Group 2 / True trees / 70 | 0.009 / 0.002 | 0.998 / 1 | 0.859 / 0.865 |
| Group 2 / 1,000bp / 70 | 0.012 / 0.003 | 0.998 / 1 | 0.833 / 0.846 |
| Group 2 / 10,000bp / 70 | 0.009 / 0.002 | 0.998 / 1 | 0.859 / 0.863 |
| Group 2 / True trees / 80 | 0.009 / 0.002 | 0.998 / 1 | 0.867 / 0.916 |
| Group 2 / 1,000bp / 80 | 0.014 / 0.004 | 0.998 / 0.999 | 0.847 / 0.857 |
| Group 2 / 10,000bp / 80 | 0.01 / 0.003 | 0.998 / 1 | 0.871 / 0.897 |
| Group 2 / True trees / 90 | 0.009 / 0.003 | 0.998 / 1 | 0.876 / 0.908 |
| Group 2 / 1,000bp / 90 | 0.015 / 0.005 | 0.997 / 0.999 | 0.855 / 0.883 |
| Group 2 / 10,000bp / 90 | 0.011 / 0.003 | 0.998 / 0.999 | 0.873 / 0.889 |

## Analysis of NGS with phyloscanner

To understand the accuracy of phyloscanner in predicting true transmission pairs, we used PRC (see Methods). Our results were consistently in showing that the performance of phyloscanner was better than a random classifier, represented by the horizontal lines in the plots. Results were similar independent of data analyzed (Figure S14).

The area under the curve (AUC) of PRC can be used as a score for comparison between PRCs. The AUC for phyloscanner analyses was an average of 69% which was higher than the AUC for PRCs reconstructed for consensus sequences (Figure S14).

**Figure S14:** **Precision-recall curves for phyloscanner analysis.** Plot showing the precision-recall curves (PRC) for phyloscanner results filtered by infector probability ≥ 1% of the diagnosed individuals not on ART for parameter groups 1 and 2 and an average of 250 and 750 migrants per year to and from *region*. The AUC for migration (mig) = 250 is 0.68 and 0.71 for parameter groups 1 and 2, respectively. The AUC for mig = 750 is 0.69 and 0.70 for parameter group 1 and 2, respectively.

Our results also showed that similarly to analysis with consensus sequences, specificity was very high which means that we could correctly identify non-transmission pairs. On the other hand, sensitivity was also low showing that phyloscanner could identify few pairs as TP compared to the large number of pairs identified as FN. The sensitivity for phyloscanner analysis was slightly higher than sensitivity estimated for consensus sequences. Finally, precision values were similar independent of data analyzed (Tables S20–S21).

**Table S20:** **Total pairs analyzed with phyloscanner:** Absolute number for true positives (TP), false positives (FP), true negatives (TN) and false negatives (FN) estimated for phyloscanner analysis. Here we quantified whether a pair represented a transmission pair independent of who infected whom filtering data by infector probability (*W*) greater than or equal to 80% or 1%

|  | **W ≥ 80% / W ≥ 1%** | | | | |
| --- | --- | --- | --- | --- | --- |
| **Parameter: Migration*** | **TP** | **FP** | **TN** | **FN** | **TN / TP sample** |
| Group 1: 250 | 280 / 808 | 43 / 238 | 4,471 / 4,276 | 3,790 / 3,262 | 4,514 / 4,070 |
| Group 2: 250 | 413 / 1,153 | 46 / 269 | 4,959 / 4,733 | 4,671 / 3,931 | 5,002 / 5,084 |
| Group 1: 500 | 212 / 697 | 34 / 156 | 3,750 / 3,628 | 3,522 / 3,037 | 3,784 / 3,734 |
| Group 2: 500 | 339 / 988 | 37 / 245 | 4,569 / 4,361 | 4,429 / 3,780 | 4,606 / 4,768 |
| Group 1: 750 | 174 / 688 | 21 / 138 | 3,667 / 3,550 | 3,526 / 3,012 | 3,688 / 3,700 |
| Group 2: 750 | 264 / 913 | 39 / 211 | 4,625 / 4,453 | 4,430 / 3,781 | 4,664 / 4,694 |

* The two combination of parameter values (group 1 and group 2) used to simulate the networks as reported in Table S5. Migration is the average number of migrants per year to and from *region*.

**Table S21:** **Phyloscanner results:** Sensitivity, specificity and precision for phyloscanner analyses. Here we quantified whether a pair represented a transmission pair independent of who infected whom filtering data by infector probability (*W*) greater than or equal to 80% or 1%.

|  | **W ≥ 80% / W ≥ 1%** | | |
| --- | --- | --- | --- |
| **Parameter: Migration*** | **Sensitivity** | **Specificity** | **Precision** |
| Combination 1: 250 | 0.07 / 0.20 | 0.99 / 0.95 | 0.87 / 0.77 |
| Combination 2: 250 | 0.08 / 0.23 | 0.99 / 0.95 | 0.90 / 0.81 |
| Combination 1: 750 | 0.05 / 0.19 | 0.99 / 0.96 | 0.89 / 0.83 |
| Combination 2: 750 | 0.06 / 0.20 | 0.99 / 0.95 | 0.87 / 0.81 |

* The two combination of parameter values used to simulate the networks as reported in Table 2 (main text). Migration is the average number of migrants per year to and from *region*.

For the pairs analyzed with phyloscanner and identified as TN, we observed an average of 79% and 65% of one intermediary ID for pairs filtered by *W*≥80% and *W*≥1%, respectively. Note that we know the intermediary IDs because we compared the results obtained with phyloscanner with the transmission network simulations. When checking the order of transmissions, most pairs (average of 87% and 88.5% for pairs filtered by *W* ≥ 80% and *W* ≥ 1%, respectively) followed a pattern of intermediary→host.1 and intermediary(s)→host.2, or intermediary→host.1 and host.2→intermediary, where host.1 and host.2 were the pair analyzed by phyloscanner. The remainder of pairs (average of 13% and 11% for pairs filtered by *W* ≥ 80% and *W* ≥ 1%, respectively) followed a pattern of host.1→intermediary→host.2. For those transmissions, we observed that the difference between the true transmission times of the two IDs identified as a pair by phyloscanner was a median of 4.16 months (CI = 1.77 to 7.05 months) and 5.42 months (CI = 2.14 to 11.11 months) for pairs filtered by *W* ≥ 80% and *W* ≥ 1%, respectively.

For an average of 250 migrants per year to and from *region*, we observed an average of 91% and 86% of one intermediate ID for pairs identified as false positives (FP) filtered by *W* ≥ 80% and *W* ≥ 1%, respectively. The order of transmissions followed a transmission chain in the form of host.1→intermediary(s)→host.2, where host.1 and host.2 were the pair analyzed by phyloscanner. The difference of the true transmission times between the two IDs identified as a pair showed a median of 3.78 months (CI = 1.77 to 6.93 months) and 3.60 months (CI = 1.47 to 7.79 months) for pairs filtered by *W* ≥ 80% and *W* ≥ 1%, respectively.

For an average of 500 migrants per year to and from *region*, we observed an average of 93% and 84% of one intermediary ID for pairs identified as FP filtered by W ≥ 80% and W ≥ 1%, respectively. The order of transmissions followed a transmission chain in the form of host.1→intermediary(s)→host.2, where host.1 and host.2 were the pair analyzed by phyloscanner. The time of each transmission involved in the transmission chain was also very short. When we checked the difference of the true transmission times between the two IDs identified as a pair (host.1 and host.2) we observed a median of 3.05 months (CI = 1.07 – 6.85 months) and 4·08 months (CI = 1.41 – 8.0 months) for pairs filtered by *W* ≥ 80% and *W* ≥ 1%, respectively.

For an average of 750 migrants per year to and from *region*, we observed an average of 87% and 85% of one intermediate ID for pairs identified as false positives filtered by *W* ≥ 80% and *W* ≥ 1%, respectively. The order of transmissions followed a transmission chain in the form of host.1→intermediary(s)→host.2, where host.1 and host.2 were the pair analyzed by phyloscanner. The difference of the true transmission times between the two IDs identified as a pair showed a median of 2.96 months (CI = 1.35 to 7.55 months) and 4.26 months (CI = 1.64 to 9.82 months) for pairs filtered by *W* ≥ 80% and *W* ≥ 1%, respectively.

# References

1 Jenness SM, Goodreau SM, Morris M. **EpiModel: An R package for mathematical modeling of infectious disease over networks**. *J Stat Softw* 2018; **84**:8.

2 Carnegie NB, Krivitsky PN, Hunter DR, Goodreau SM. **An approximation method for improving dynamic network model fitting**. *J Comput Graph Stat Jt Publ Am Stat Assoc Inst Math Stat Interface Found N Am* 2015; **24**:502–519.

3 Krivitsky PN, Handcock MS. **A separable model for dynamic networks**. *J R Stat Soc Ser B Stat Methodol* 2014; **76**:29–46.

4 Krivitsky PN, Handcock MS, Morris M. **Adjusting for network size and composition effects in exponential-family random graph model**. *Stat Methodol* 2011; **8**:319–339.

5 Jenness SM, Goodreau, SM, Weiss K. EpiModelHIV: Network-based epidemic modeling of HIV transmission among MSM and heterosexual populations. R package. 2017.

6 Weiss KM, Goodreau SM, Morris M, Prasad P, Ramaraju R, Sanchez T, *et al.* **Egocentric sexual networks of men who have sex with men in the United States: Results from the ARTnet study**. *Epidemics* 2020; **30**:100386.

7 Pines HA, Karris MY, Little SJ. **Sexual partner concurrency among partners reported by MSM with recent HIV infection**. *AIDS Behav* 2017; **21**:3026–3034.

8 Le Vu S, Ratmann O, Delpech V, Brown AE, Gill ON, Tostevin A, *et al.* **Comparison of cluster-based and source-attribution methods for estimating transmission risk using large HIV sequence databases**. *Epidemics* 2018; **23**:1–10.

9 Grey JA, Bernstein KT, Sullivan PS, Purcell DW, Chesson HW, Gift TL, *et al.* **Estimating the population sizes of men who have sex with men in US states and counties using data from the American community survey**. *JMIR Public Health Surveill* 2016; **2**:e14.

10 Nascimento FF. Analyses of transmission network data: Supplementary tables. 2022.https://github.com/thednainus/HIVepisimAnalysis/blob/main/Manuscript (accessed 22 Sep2023).

11 Cori A, Pickles M, van Sighem A, Gras L, Bezemer D, Reiss P, *et al.* **CD4+ cell dynamics in untreated HIV-1 infection: overall rates, and effects of age, viral load, sex and calendar time**. *AIDS Lond Engl* 2015; **29**:2435–2446.

12 van Sighem A, Nakagawa F, De Angelis D, Quinten C, Bezemer D, de Coul EO, *et al.* **Estimating HIV incidence, time to diagnosis, and the undiagnosed HIV epidemic using routine surveillance data**. *Epidemiol Camb Mass* 2015; **26**:653–660.

13 Pinkerton SD. **HIV transmission rate modeling: a primer, review, and extension**. *AIDS Behav* 2012; **16**:791–796.

14 Stein M. **Large sample properties of simulations using Latin hypercube sampling**. *Technometrics* 1987; **29**:143–151.

15 Carnell R. lhs: Latin Hypercube Samples. R package. 2022.

16 Ratmann O, Hodcroft EB, Pickles M, Cori A, Hall M, Lycett S, *et al.* **Phylogenetic tools for generalized HIV-1 epidemics: Findings from the PANGEA-HIV methods comparison**. *Mol Biol Evol* 2017; **34**:185–203.

17 Ratmann O, Hodcroft EB, Pickles M, Cori A, Hall M, Lycett S, *et al.* **Phylogenetic tools for generalized HIV-1 epidemics: Findings from the PANGEA-HIV methods comparison**. *Mol Biol Evol* 2017; **34**:185–203.

18 Rambaut A, Grassly NC. **Seq-Gen: an application for the Monte Carlo simulation of DNA sequence evolution along phylogenetic trees**. *Comput Appl Biosci CABIOS* 1997; **13**:235–238.

19 Hasegawa M, Kishino H, Yano T. **Dating of the human-ape splitting by a molecular clock of mitochondrial DNA**. *J Mol Evol* 1985; **22**:160–174.

20 Patiño-Galindo JÁ, González-Candelas F. **The substitution rate of HIV-1 subtypes: a genomic approach**. *Virus Evol* 2017; **3**:vex029.

21 Minh BQ, Schmidt HA, Chernomor O, Schrempf D, Woodhams MD, von Haeseler A, *et al.* **IQ-TREE 2: New models and efficient methods for phylogenetic inference in the genomic era**. *Mol Biol Evol* 2020; **37**:1530–1534.

22 Yang Z. **Among-site rate variation and its impact on phylogenetic analyses**. *Trends Ecol Evol* 1996; **11**:367–372.

23 Volz EM, Frost SDW. **Scalable relaxed clock phylogenetic dating**. *Virus Evol* 2017; **3**:vex025.

24 Volz E. phydynR: Phylogenetic dating and phylodynamic inference by sequential Monte Carlo. https://github.com/emvolz-phylodynamics/phydynR (accessed 22 Jan2019).

25 Huang W, Li L, Myers JR, Marth GT. **ART: a next-generation sequencing read simulator**. *Bioinforma Oxf Engl* 2012; **28**:593–594.

26 Zhang Y, Wymant C, Laeyendecker O, Grabowski MK, Hall M, Hudelson S, *et al.* **Evaluation of phylogenetic methods for inferring the direction of human immunodeficiency virus (HIV) transmission: HIV prevention trials network (HPTN) 052**. *Clin Infect Dis* 2021; **72**:30–37.
